# Supplementary material for: Crystal structure generation with autoregressive large language modeling
Source: Nat Commun. 2024 Dec 6;15:10570. doi: 10.1038/s41467-024-54639-7 (PMC11624194; doi:10.1038/s41467-024-54639-7)
Supplement: Supplementary file 1 — Supplementary Information [file 41467_2024_54639_MOESM1_ESM.pdf]

Supplementary Information for the article "Crystal Structure Generation with Autoregressive Large Language Modeling"

Luis M. Antunes[1], Keith T. Butler[2], and Ricardo Grau-Crespo[1]

[1]*Department of Chemistry, University of Reading, Whiteknights, Reading RG6 6DX, United Kingdom.*
l.m.antunes@pgr.reading.ac.uk
[2]*Department of Chemistry, University College London, WC1H0AJ, United Kingdom.*

Supplementary Notes

1. CIF Syntax Standardization and Tokenization

The CIF format is flexible in terms of the sequence of tags in the file. Moreover, not all tags are required to be present in the file. While a large language model could, in principle, learn to process variable arrangements of the tags, we chose to restrict the CIF file syntax, such that every CIF file in the dataset is structured identically. Furthermore, we added several tags that are not part of the CIF specification.

To ensure consistency, and enhance the model's ability to learn from the data, we standardized the CIF files using a sequence of pre-processing steps. The steps were designed to not only normalize the format of the CIF files, but also to incorporate additional information beneficial for the model's training. The pre-processing steps are as follows:

1. Each structure in the dataset was first converted into a pymatgen `Structure` object.

2. The pymatgen `CifWriter` class was used to create CIF files from the `Structure` objects, using a `symprec` value of 0.1.

3. In the created CIF files, we replaced the content of the `data_` tag, which contains the reduced formula, with the cell composition of the structure. The atoms of the cell composition appended to `data_` are sorted by electronegativity.

4. We removed the symmetry operators from the CIF files.

5. We introduced a custom block in the CIF files to include specific atomic properties, namely, the electronegativity, the radius, and ionic radius. These properties are not part of the standard CIF specification.

6. All numerical values in the CIF files were rounded to four decimal places.

An example of a CIF file from the training dataset, both before and after it has been pre-processed, is given below:

Original CIF file for PbTe (Z=2, Pmma), before pre-processing

```
data_TePb
_symmetry_space_group_name_H-M   Pmma
_cell_length_a   5.64400000
_cell_length_b   4.00120000
_cell_length_c   5.68070000
_cell_angle_alpha   90.00000000
_cell_angle_beta   90.00000000
_cell_angle_gamma   90.00000000
_symmetry_Int_Tables_number   51
_chemical_formula_structural   TePb
_chemical_formula_sum   'Te2 Pb2'
_cell_volume   128.28595744
_cell_formula_units_Z   2
loop_
 _symmetry_equiv_pos_site_id
 _symmetry_equiv_pos_as_xyz
  1  'x, y, z'
  2  '-x, -y, -z'
  3  '-x+1/2, -y, z'
  4  'x+1/2, y, -z'
  5  'x+1/2, -y, -z'
  6  '-x+1/2, y, z'
  7  '-x, y, -z'
  8  'x, -y, z'
loop_
 _atom_site_type_symbol
 _atom_site_label
 _atom_site_symmetry_multiplicity
 _atom_site_fract_x
 _atom_site_fract_y
 _atom_site_fract_z
 _atom_site_occupancy
  Te  Te0  2  0.25000000  0.50000000  0.73570000  1.0
  Pb  Pb1  2  0.25000000  0.00000000  0.26910000  1.0
```

Pre-processed CIF file for PbTe (Z=2, Pmma)

```
data_Te2Pb2
loop_
_atom_type_symbol
_atom_type_electronegativity
_atom_type_radius
_atom_type_ionic_radius
Te 2.1000 1.4000 1.2933
Pb 2.3300 1.8000 1.1225
_symmetry_space_group_name_H-M Pmma
_cell_length_a 5.6440
_cell_length_b 4.0012
_cell_length_c 5.6807
_cell_angle_alpha 90.0000
_cell_angle_beta 90.0000
_cell_angle_gamma 90.0000
_symmetry_Int_Tables_number 51
```

```
_chemical_formula_structural TePb
_chemical_formula_sum 'Te2 Pb2'
_cell_volume 128.2864
_cell_formula_units_Z 2
loop_
_symmetry_equiv_pos_site_id
_symmetry_equiv_pos_as_xyz
1 'x, y, z'
loop_
_atom_site_type_symbol
_atom_site_label
_atom_site_symmetry_multiplicity
_atom_site_fract_x
_atom_site_fract_y
_atom_site_fract_z
_atom_site_occupancy
Te Te0 2 0.2500 0.5000 0.7357 1
Pb Pb1 2 0.2500 0.0000 0.2691 1
```

After the pre-processing step, the CIF files are tokenized; that is, a CIF file is parsed and converted into a sequence of numbers, where each number represents a particular token. Tokenization is necessary for converting the structured, text-based data of CIF files into a format that the model can process. The selection of tokens is guided by a custom vocabulary. The vocabulary defines the distinct, irreducible elements of the CIF file syntax that are relevant to the problem. In constructing this vocabulary, we chose to represent numeric digits, atomic symbols, space group symbols, and CIF tags with distinct tokens. Specifically, the vocabulary consists of digits: `0 1 2 3 4 5 6 7 8 9`, as well as various symbols: `x y z . ( ) ' ,` ␣ (space) \n (newline). A complete enumeration of the supported atom, CIF tag, and space group symbols follows:

Supported atom tokens.

```
Ac Ag Al Ar As Au B Ba Be Bi Br C Ca Cd Ce Cl Co Cr Cs Cu Dy Er Eu F Fe Ga Gd Ge
H He Hf Hg Ho I In Ir K Kr La Li Lu Mg Mn Mo N Na Nb Nd Ne Ni Np O Os P Pa Pb Pd
Pm Pr Pt Pu Rb Re Rh Ru S Sb Sc Se Si Sm Sn Sr Ta Tb Tc Te Th Ti Tl Tm U V W Xe
Y Yb Zn Zr
```

Supported CIF tag tokens. Tags with * do not exist in the official CIF specification.

_cell_length_b	_atom_site_occupancy
_atom_site_attached_hydrogens	_cell_length_a
_cell_angle_beta	_symmetry_equiv_pos_as_xyz
_cell_angle_gamma	_atom_site_fract_x
_symmetry_space_group_name_H-M	_symmetry_Int_Tables_number
_chemical_formula_structural	_chemical_name_systematic
_atom_site_fract_y	_atom_site_symmetry_multiplicity
_chemical_formula_sum	_atom_site_label
_atom_site_type_symbol	_cell_length_c
_atom_site_B_iso_or_equiv	_symmetry_equiv_pos_site_id
_cell_volume	_atom_site_fract_z
_cell_angle_alpha	_cell_formula_units_Z
loop_	data_
_atom_type_symbol	_atom_type_electronegativity *
_atom_type_radius *	_atom_type_ionic_radius *
_atom_type_oxidation_number	

Supported space group tokens.

Aea2	Aem2	Ama2	Amm2	C2	C2/c	C2/m
C222	C222_1	Cc	Ccc2	Ccce	Cccm	Cm
Cmc2_1	Cmce	Cmcm	Cmm2	Cmme	Cmmm	F-43c
F-43m	F222	F23	F432	F4_132	Fd-3	Fd-3c
Fd-3m	Fdd2	Fddd	Fm-3	Fm-3c	Fm-3m	Fmm2
Fmmm	I-4	I-42d	I-42m	I-43d	I-43m	I-4c2
I-4m2	I222	I23	I2_12_12_1	I2_13	I4	I4/m
I4/mcm	I4/mmm	I422	I432	I4_1	I4_1/a	I4_1/acd
I4_1/amd	I4_122	I4_132	I4_1cd	I4_1md	I4cm	I4mm
Ia-3	Ia-3d	Iba2	Ibam	Ibca	Im-3	Im-3m
Ima2	Imm2	Imma	Immm	P-1	P-3	P-31c
P-31m	P-3c1	P-3m1	P-4	P-42_1c	P-42_1m	P-42c
P-42m	P-43m	P-43n	P-4b2	P-4c2	P-4m2	P-4n2
P-6	P-62c	P-62m	P-6c2	P-6m2	P1	P2
P2/c	P2/m	P222	P222_1	P23	P2_1	P2_1/c
P2_1/m	P2_12_12	P2_12_12_1	P2_13	P3	P312	P31c
P31m	P321	P3_1	P3_112	P3_121	P3_2	P3_212
P3_221	P3c1	P3m1	P4	P4/m	P4/mbm	P4/mcc
P4/mmm	P4/mnc	P4/n	P4/nbm	P4/ncc	P4/nmm	P4/nnc
P422	P42_12	P4_1	P4_122	P4_12_12	P4_132	P4_2
P4_2/m	P4_2/mbc	P4_2/mcm	P4_2/mmc	P4_2/mnm	P4_2/n	P4_2/nbc
P4_2/ncm	P4_2/nmc	P4_2/nnm	P4_22_12	P4_232	P4_2bc	P4_2cm
P4_2mc	P4_2nm	P4_3	P4_322	P4_32_12	P4_332	P4bm
P4cc	P4mm	P4nc	P6/m	P6/mcc	P6/mmm	P622
P6_1	P6_122	P6_2	P6_222	P6_3	P6_3/m	P6_3/mcm
P6_3/mmc	P6_322	P6_3cm	P6_3mc	P6_4	P6_422	P6_5
P6_522	P6cc	P6mm	Pa-3	Pba2	Pbam	Pban
Pbca	Pbcm	Pbcn	Pc	Pca2_1	Pcc2	Pcca
Pccm	Pccn	Pm	Pm-3	Pm-3m	Pm-3n	Pma2
Pmc2_1	Pmm2	Pmma	Pmmm	Pmmn	Pmn2_1	Pmna
Pn-3	Pn-3m	Pn-3n	Pna2_1	Pnc2	Pnma	Pnn2
Pnna	Pnnm	Pnnn	R-3	R-3c	R-3m	R3
R32	R3c	R3m				

The atom tokens cover all 89 atom types present in the training data. Atoms with atomic number $Z \geq 84$ (Po) are thus excluded (except for the early actinides Ac, Th, Pa, U, Np, and Pu, which did appear in crystal structures in the databases).

The 227 space group symbols also cover all space groups present in the training data. The space groups $P4_222$ (no. 93), P6 (no. 168), and P432 (no. 207) are not supported as there are no structures in the training data with these space groups. These three space groups are known to occur very rarely, due to a combination of symmetries (and absences of symmetries) that are difficult to realise in a crystal geometry. [1] For example, the space group P6 requires the presence of a six-fold rotation axis but without the presence of mirror planes and inversion centres that occur in other hexagonal groups. Note that the rarity of these space groups is not limited to the ab initio databases used in our study. The ICSD database, which lists crystallographic information for most currently known materials and minerals, does not contain any experimentally-determined ordered inorganic compounds in these three space groups (only three experimental inorganic crystal structures are listed for these space groups: $K_2Ta_4O_9F_4$ and $MoCu_2Al_{7.92}$ for space group P6, and $Rb(NO_3)$ for space group P432, but they all exhibit site-occupancy disorder).

After the model has generated a sequence of tokens representing a CIF file, we perform a post-processing step in which the custom `loop_` section with atomic properties is

removed, and the symmetry equivalent site IDs and positions which match the printed space group are introduced.

2. Model Architecture and Generative Pre-training

The generative pre-training step consists of training a GPT-style transformer model autoregressively. The implementation is based on the nanoGPT project [2]. The model consists of a series of transformer blocks, each consisting of multi-head self-attention and a feed-forward neural network. The input to the model is a sequence of token indices representing the token sequence. The tokens are embedded using a learned embedding table. The token embeddings are combined with learned positional embeddings, to which dropout is applied. The result is passed through a series of transformer blocks. A transformer block consists of causal self-attention [3] and a feed-forward network containing a non-linear layer with GELU activation [4], and dropout. A linear output layer transforms the features produced by the transformer blocks into a vector of logits. A softmax operation is applied to convert the logits into the probabilities of the tokens of the vocabulary, for each position in the output sequence. Weight tying [5] is used: the output layer and the input embedding layer share the same weights. The objective is to minimize the cross-entropy loss between the predicted probability distribution over the vocabulary and the actual next token in the sequence, for all the tokens in the sequence.

Training consists of iteratively sampling sequences from the dataset, performing a forward-pass through the model, computing the loss, and backpropagating the error. We use the AdamW optimizer [6], and apply a cosine decay schedule to the learning rate, from 10^{-3} to 10^{-4}, over the course of training. Gradients were clipped to have a norm of at most 1.0. During each training iteration, we perform 40 gradient accumulation steps, and in each step we randomly sample a batch of 32 sequences. The dataset consists of a single list of all tokens from all CIF files, concatenated together, and the beginning of each sequence is a randomly sampled token from the list. The number of tokens in each sequence is equal to the block size of the model, which is the maximum length of the input sequence the model can process. All models were trained on a single A100 GPU with 80 GB of memory.

2.1 Small Model

The small model consists of 25 million parameters, with 8 transformer blocks, each with 8 attention heads, an embedding size of 512, a block size of 1,024, and dropout with probability $p = 0.1$. To determine the optimal number of training iterations, we train the model using 10% of the dataset as a validation set, and monitor the model's performance on the validation set, in terms of the cross-entropy loss. It was determined that the model continues to improve beyond 90,000 iterations. Therefore, the final model was trained on the entire dataset for 100,000 iterations (due to computational resource and time constraints).

2.2 Large Model

The large model consists of 200 million parameters, with 16 transformer blocks, each with 16 attention heads, an embedding size of 1,024, a block size of 2,048, and dropout

with probability $p = 0.1$. Due to computational resource and time constraints, we train the large model on the entire dataset for 48,000 iterations. Additionally, the starting point for each sequence is sampled from a pre-compiled list of tokens, each known to be the starting token of a CIF file in the dataset. This approach ensures that each sequence begins at the start of a distinct CIF file.

2.3 Training Times

On an A100 GPU, the small model requires 3 seconds per training iteration. Therefore, 100,000 small model training iterations requires 83.3 hours, or approximately 3.5 days. The large model requires 16 seconds per training iteration on an A100 GPU. Therefore, 48,000 large model training iterations requires 213.3 hours, or approximately 8.9 days.

2.4 CIF File Generation via Random Sampling

CIF files are generated using top-k random sampling. Top-k sampling involves randomly selecting the next token from the top k most likely candidates as predicted by the model. We first apply temperature scaling $\mathbf{x}/\tau$ to the logits $\mathbf{x} \in \mathbb{R}^{|\mathcal{V}|}$ at the final position, and keep only the top k logits, where $|\mathcal{V}|$ represents the size of the vocabulary. The top k logits are then converted into normalized probabilities through application of the softmax operation. Finally, the next token is sampled using the given probabilities. More formally,

$$\text{token} \sim \text{softmax}\left(\text{top}_k\left(\frac{\mathbf{x}}{\tau}\right)\right) \tag{1}$$

$$\text{softmax}(\mathbf{x})_i = \frac{e^{x_i}}{\sum_{j=1}^{k} e^{x_j}} \tag{2}$$

where i represents the i-th element of the vector $\mathbf{x}$. Tokens are sampled iteratively, each conditioned on the progressively growing sequence of previously sampled tokens, until two consecutive newline tokens are sampled.

3. Validation of Generated CIF Files

To ensure the consistency of the printed information and the chemical sensibility of the implied structure, we conduct a series of validations on the generated CIF file. The procedure is described in Algorithm 1.

First, we require that the chemical formula, which is printed in several locations in the file, is consistent everywhere. Specifically, we ensure that the formula associated with the `_chemical_formula_sum` tag, and the (reduced) formula associated with the `_chemical_formula_structural` tag are consistent with the cell composition in the first line of the file, and with each other.

Next, we require that the printed atom site multiplicity is consistent with the cell composition. This information is printed at the end of the file, and the values are associated with the `_atom_site_type_symbol` and `_atom_site_symmetry_multiplicity` tags.

We also check that the structure's bond lengths are reasonable. To check if bond lengths are reasonable, we first use a Voronoi-based nearest-neighbour algorithm in pymatgen to define which atoms are bonded together; then, we establish expected bond lengths based on the electronegativity difference between the bonded atoms, and their ionic or covalent radii. We compute a bond length reasonableness score, $B \in [0, 1]$, which represents the fraction of bonds which are within 30% of the corresponding expected bond lengths. We classify a structure as having reasonable bond lengths if $B \geq c_{\text{bond}}$, where $c_{\text{bond}} \in [0, 1]$ is a bond length acceptability score minimum, which in this work is set to 1.0 (i.e. all bond lengths must be within 30% of the expected bond lengths).

Finally, we check that the generated CIF file is consistent in terms of space group. To check if the generated structure is consistent with the printed space group, we use the `SpacegroupAnalyzer` class of the pymatgen library, which uses the spglib library [7].

Algorithm 1 Check Validity of Generated CIF File

```
Input: S, the contents of the generated CIF file
Input: c_bond, the bond length acceptability score minimum
Output: True or False, indicating whether S is valid
if not is_formula_consistent(S) then
    return False
end if
if not is_atom_site_multiplicity_consistent(S) then
    return False
end if
B ← bond_length_reasonableness_score(S)
if B < c_bond then
    return False
end if
if not is_space_group_consistent(S) then
    return False
end if
return True
```

4. Monte Carlo Tree Search Decoding

To improve the efficiency and quality of sampling from the model, we use the Monte Carlo Tree Search (MCTS) algorithm [8, 9]. Typically, the MCTS algorithm is used in the context of games, and similar decision processes, where the aim is to select an optimal action to perform. Here, we use MCTS to generate a collection of sequences, which should improve (according to some measure of quality) as the algorithm proceeds.

A sequence of tokens can be considered the outcome of following a specific path during the traversal of a tree of tokens, starting from the root and progressing to a leaf. In this framework, each node in the tree represents a token, and each edge denotes the transition from one token to the next in the sequence. By systematically exploring and expanding the most promising paths, MCTS balances exploitation of well-performing token sequences with exploration of new, potentially better sequences. The trade-off between exploitation and exploration is achieved through a principled selection strategy,

which guides the search towards areas of the tree that either have high potential or have not been sufficiently explored. As the search progresses, the algorithm builds a more informed representation of the tree, enabling more efficient and higher-quality sampling of token sequences.

The MCTS algorithm is comprised of a sequence of steps performed for a fixed number of iterations. Our implementation is described in Algorithm 2, and a detailed explanation of each step follows.

4.1 Selection

Each node, i, in the tree represents the cumulative context up to that point, akin to constructing a sentence word by word. The first step in every iteration involves descending the tree, from the root node to a leaf node, by selecting the most promising node, i_t, at each level t. To select the node, we use a variant of the PUCT (Predictor-Upper Confidence bound applied to Trees) algorithm [10, 11].

The selection of a node at each level is guided by the statistics accumulated in the tree. The specific node i_t is chosen by maximizing the PUCT value, expressed as $i_t = \underset{i}{\operatorname{argmax}}(\text{PUCT}(i_t))$, where the PUCT value is calculated as:

$$\text{PUCT}(i) = \frac{w_i}{N_i} + c_{\text{puct}} P_i \frac{\sqrt{N_h}}{1 + N_i} \tag{3}$$

where w_i represents the total score accumulated at node i, indicating the node's past performance. N_i is the number of times node i has been visited, reflecting its exploitation level. P_i is the prior probability of selecting the token leading to node i, given its parent node h, which is provided by the CrystaLLM model. N_h is the total number of visits to the parent node h, and c_{puct} is a constant determining the level of exploration in the PUCT algorithm.

4.2 Expansion

If a node has children that haven't been added to the tree, a child node is selected randomly and added to the tree. This newly added node is the selected node for the remainder of the iteration. To determine what children a node contains, the CrystaLLM model's predicted probabilities are used to select the top k tokens. If a child node's probability exceeds 0.99, it becomes the sole child node. In this case, where a node has only a single child node, the child node is bypassed, foregoing the Rollout step. The process then proceeds directly to the Selection step for the child nodes of the bypassed node, continuing the iteration from that point onwards.

4.3 Rollout

The Rollout step involves prompting the CrystaLLM model with the sequence of tokens represented by the selected node in the tree, and then sampling from the model repeatedly, until a terminating condition is reached. The aim is to arrive at a completed structure, which can then be further evaluated and scored.

4.4 Evaluation

Once a sequence has been completed, either by reaching a terminal node through Selection, or through Rollout, it represents the contents of a completed CIF file. The generated CIF contents are then validated (see Supplementary Note 3), and if the generated CIF file is valid, the structure is evaluated using the ALIGNN model of formation energy per atom, to produce a prediction of the structure's energy, E_{f}.

4.5 Backpropagation

The outcomes of iterations are accumulated in the tree nodes. All nodes selected during a simulation increase in their visit count, and a score is added to each. The score, $R \in [-1, 1]$, represents the quality of the generated structure. A more positive R represents a better structure.

Because scores are required to be between -1 and 1, and since the range of formation energies is not known for a composition a priori, the score for valid structures ($R_{\text{valid}} \in [0, 1]$) is computed using the statistics of the predicted energies over the course of the search:

$$R_{\text{valid}} = \frac{1}{1 + e^{\lambda((E_{\mathrm{f}} - \mu)/\sigma)}} \tag{4}$$

where E_{f} is the formation energy per atom (eV) according to ALIGNN (Atomistic Line Graph Neural Network) [12], μ is the mean over all of the obtained E_{f}, σ is the standard deviation over all the obtained E_{f}, and λ is a constant that determines how responsive the reward is to E_{f}.

The overall score is computed piecewise:

$$R = \begin{cases} R_{\text{valid}} & \text{if valid,} \\ B - 1 & \text{if bond lengths unreasonable,} \\ -1 & \text{otherwise} \end{cases} \tag{5}$$

where B is the bond length reasonableness score. An invalid structure receives a score of -1, unless it is invalid because of unreasonable bond lengths. In cases where a CIF file is otherwise valid, but the structure contains unreasonable bond lengths, a negative score is assigned that is proportional to the number of unreasonable bonds.

Algorithm 2 Monte Carlo Tree Search Decoding

```
Input: trained large language model, LLM
Input: number of simulations, n
Input: tree width, k
Input: PUCT exploration constant, c_puct
Input: text prompt, P
Output: list of valid sequences
Initialize tree with root node based on P
valid_sequences ← []
for simulation = 1 to n do
    current_node ← root
    // Select
    while not current_node.has_untried_children() and current_node.has_children() do
        current_node ← select_node(current_node.children, LLM, c_puct)
    end while
    // Expand
    if current_node.has_untried_children() then
        untried_child ← select_untried_child_randomly(current_node, k)
        current_node.add_child(untried_child)
        current_node ← untried_child
    end if
    // Rollout
    complete_sequence ← sample_randomly(current_node, LLM)
    // Evaluate
    score ← evaluate_sequence(complete_sequence)
    if is_valid(complete_sequence) then
        valid_sequences.append(complete_sequence)
    end if
    // Backpropagate
    while current_node is not null do
        current_node.visits ← current_node.visits + 1
        current_node.wins ← current_node.wins + score
        current_node ← current_node.parent
    end while
end for
return valid_sequences
```

5. Metrics for Unconditional Generation

To assess performance on the unconditional generation tasks, we use the metrics introduced by Xie et al. [13]. Six metrics are used to evaluate different aspects of generation quality: COV-R (coverage recall), COV-P (coverage precision), AMSD-R (average minimum structure distance recall), AMSD-P (average minimum structure distance precision), AMCD-R (average minimum composition distance recall) and AMCD-P (average minimum composition distance precision).

Using the same formalism introduced by [13], we re-state here the definitions of these metrics: We would like to compare a collection of K materials generated by a model, $\{M_k\}_{k\in[1..K]}$, to a collection of L ground-truth materials, $\{M^*{}_l\}_{l\in[1..L]}$. We further define a distance between two structures of the collections, $D_{\text{struc}}(M_k, M^*{}_l)$, and a distance between two compositions of the collections, $D_{\text{comp}}(M_k, M^*{}_l)$. For all metrics, the structure distance is the Euclidean distance between the CrystalNN fingerprints [14] for the two structures, while the composition distance is the Euclidean distance between the normalized Magpie fingerprints [15] of the two compositions. Moreover, thresholds $\delta_{\text{struc}}, \delta_{\text{comp}} \in \mathbb{R}$ are defined for the structure and composition distances.

The metrics are thus defined as follows:

$$\begin{aligned}\text{COV-R} = \frac{1}{L}|\{l \in [1..L] : \exists k \in [1..K], D_{\text{struc}}(M_k, M^*{}_l) < \delta_{\text{struc}},\\ D_{\text{comp}}(M_k, M^*{}_l) < \delta_{\text{comp}}\}|\end{aligned} \tag{6}$$

$$\begin{aligned}\text{COV-P} = \frac{1}{K}|\{k \in [1..K] : \exists l \in [1..L], D_{\text{struc}}(M_k, M^*{}_l) < \delta_{\text{struc}},\\ D_{\text{comp}}(M_k, M^*{}_l) < \delta_{\text{comp}}\}|\end{aligned} \tag{7}$$

$$\text{AMSD-R} = \frac{1}{L}\sum_{l\in[1..L]} \min_{k\in[1..K]} D_{\text{struc}}(M_k, M^*{}_l) \tag{8}$$

$$\text{AMSD-P} = \frac{1}{K}\sum_{k\in[1..K]} \min_{l\in[1..L]} D_{\text{struc}}(M_k, M^*{}_l) \tag{9}$$

$$\text{AMCD-R} = \frac{1}{L}\sum_{l\in[1..L]} \min_{k\in[1..K]} D_{\text{comp}}(M_k, M^*{}_l) \tag{10}$$

$$\text{AMCD-P} = \frac{1}{K}\sum_{k\in[1..K]} \min_{l\in[1..L]} D_{\text{comp}}(M_k, M^*{}_l) \tag{11}$$

In summary, the recall metrics assess the proportion of actual materials correctly identified, whereas the precision metrics evaluate the quality of the materials generated. See [13] and [16] for more detailed discussion and description of these metrics. As in [13], we use $\delta_{\text{struc}} = 0.2$, $\delta_{\text{comp}} = 4$ for Perov-5 and Carbon-24, and $\delta_{\text{struc}} = 0.4$, $\delta_{\text{comp}} = 10$ for MP-20.

6. Effect of MCTS on the Stability of Unconditionally Generated Novel Structures

The MCTS procedure can be used to find structures with lower energy for the unconditionally generated compositions, using the ALIGNN energy evaluator as a fast proxy for DFT energies. As mentioned in the article, we performed MCTS, with 1,000 iterations, on each of the 102 compounds initially identified as novel.

The raw results are provided as a separate CSV file (see Supplementary Dataset 1), and discussed in more detail here. The MCTS procedure works as intended, lowering (or keeping constant) the ALIGNN energies of all tested compositions. The average ALIGNN

energy change is -153 ± 15 meV/atom (with the error bar obtained as the standard error of the mean). However, the mean E_{hull}, as calculated by DFT, is not reduced by the same amount, because of the limitations in accuracy of the ALIGNN energy estimator (and the fact that ALIGNN predicts energies for the as-generated, unrelaxed compounds, while the DFT energies are obtained for relaxed geometries). The MCTS-induced improvement of the average DFT energy, of -56 ± 15 meV/atom, reduces the average E_{hull} from 0.40 to 0.34 eV/atom. The question, then, is whether, given the sample size, this DFT energy lowering is still significant, or simply due to a statistical fluctuation. In other words, is the improvement introduced by the MCTS procedure in terms of ALIGNN energies maintained (with statistical significance) after evaluating the energies with DFT?

To precisely answer this question, we performed a statistical test of significance. The null hypothesis is that MCTS does not bring any improvement to the average E_{hull} obtained by DFT. In that case, the DFT energy changes (from the original structures to those generated by MCTS) would be just randomly distributed with zero mean. We can calculate what the probability p would be of obtaining our DFT results under the null hypothesis conditions. Using a paired t-test, we obtained t=-3.7, which means that the probability of the DFT energy change observed being a statistical fluctuation in either direction (two-sided test) is p=0.0003. This is well below the threshold of p=0.05 typically accepted for statistical significance. We have checked that a Wilcoxon signed-rank test, that accounts for deviations of the distribution of paired differences from normality, gives a similar result. Therefore, we can definitely reject the null hypothesis: the advantage introduced by MCTS does survive the transition from ALIGNN to DFT energies, despite the limitations of ALIGNN. Not only are the ALIGNN energies improved by the MCTS approach, but the DFT E_{hull} energies are as well.

Supplementary Tables

Supplementary Table 1: The compounds of the Challenge Set, their sources, and their formation energies per atom (E_f), as predicted by the ALIGNN model.

Formula	Source	ALIGNN E_f (eV/atom)
Ba_2MnCr	training set	0.906
$Ca_{10}(PO_4)_6(OH)_2$	training set	-3.029
$CH_3NH_3PbI_3$	training set	-0.358
$Co_2CO_3(OH)_2$	training set	-1.019
$CsCuTePt$	training set	0.137
$Cu_2C_1O_5H_2$	training set	-0.923
$Cu_3(CO_3)_2(OH)_2$	training set	-0.999
K_2AgMoI_6	training set	-0.639
MgF_2	training set	-3.782
$Mn_4(PO_4)_3$	training set	-2.010
$PbCu(OH)_2SO_4$	training set	-1.160
Sm_2BO_4	training set	-2.993
$AlCu_2As(HO)_{12}$	ref. [17]	-1.185
Ba_2AuIO_6	ref. [18]	-1.329
$Ba_2Fe_2F_9$	ref. [19]	-3.073
$Ba_6Fe_2Te_3S_7$	ref. [20]	-1.593
$Ba_2Gd(BO_3)_2F$	ref. [21]	-3.325
$Ba_4GeSb_2Se_{11}$	ref. [22]	-1.136
Ba_3GeTeS_4	ref. [23]	-1.721
Ba_2HfF_8	ref. [24]	-4.177
$BaY_{16}Si_4O_{33}$	ref. [25]	-3.702
$Ba_9Yb_2(SiO_4)_6$	ref. [26]	-3.245
$Ca_2Bi_2O_7$	ref. [27]	-1.952
$CaFe_6Ge_6$	ref. [28]	-0.200
$CaHPO_3$	ref. [29]	-2.445
$CaPt_4P_6$	ref. [30]	-0.826
$Ca_2Te_3O_8$	ref. [31]	-1.911
$CaZnV_2O_6$	ref. [32]	-2.573
$Ce_6Cd_{23}Te$	ref. [33]	-0.295
$Cs_2Al_2O_3F_2$	ref. [34]	-3.116
$Cs_8Cu_3Si_{14}O_{35}$	ref. [35]	-2.561
$Cs_3LuSi_3O_9$	ref. [36]	-2.970
$Cu_4FeGe_2S_7$	ref. [37]	-0.368
$Eu_2FeGe_2OS_6$	ref. [38]	-1.265
HgB_2S_4	ref. [39]	-0.340
$Ho_2Ir_3Si_5$	ref. [40]	-0.885
$KScP_2O_7$	ref. [41]	-2.801
$K_2Sr_4(PO_3)_{10}$	ref. [42]	-2.626
$K_6Zn(CO_3)_4$	ref. [43]	-1.817
$La_4Ga_2S_8O_3$	ref. [44]	-2.171
$LaScSe_3$	ref. [45]	-1.912
$Li_9Al_4Sn_5$	ref. [46]	-0.173
$LiBa_2AlO_4$	ref. [47]	-2.965
Li_2GeS_3	ref. [48]	-0.989
$LiMnBi$	ref. [49]	-0.004
$LiTa_2NiSe_5$	ref. [50]	-0.842
$Mg_7Pt_4Ge_4$	ref. [51]	-0.676
$NaGdSi_2O_6$	ref. [52]	-3.074
$Na_2Hf(BO_3)_2$	ref. [53]	-2.834
$Na_6Li_4WO_4(CO_3)_4$	ref. [54]	-2.010
$NaMgV_5(H_5O_6)_4$	ref. [55]	-1.540
$Na_5Mn_4P_4H_4(O_9F_2)_2$	ref. [56]	-2.140
$NaSbSe_2O_7$	ref. [57]	-1.213
$NaSb_2TeO_7$	ref. [57]	-1.502
$Na_4Sn_2Ge_5O_{16}$	ref. [58]	-1.862
$Na_3Te_2(FeO_4)_3$	ref. [59]	-1.430
$Nd_3BSi_2O_{10}$	ref. [60]	-3.352
$Ni_3Te_2O_2(PO_4)_2(OH)_4$	ref. [61]	-1.302
$RbNiFe(PO_4)_2$	ref. [62]	-1.930
Rb_3SnCl_7	ref. [63]	-1.434
$Sr_2Bi_2O_7$	ref. [27]	-1.963
$SrCo_4(OH)(PO_4)_3$	ref. [64]	-1.861
$Sr(ClO_4)_2$	ref. [65]	-0.593
$Sr_6Ge_3OSe_{11}$	ref. [66]	-1.145
$Tb_3S_3BO_3$	ref. [67]	-2.857
Tb_3TeBO_9	ref. [68]	-2.433
$YbMn_6Sn_6$	ref. [69]	-0.070
$Zn_2(HTeO_3)(AsO_4)$	ref. [70]	-1.290
Zn_2BS_3Br	ref. [71]	-0.692
$Zn_4CuH_6(CO_6)_2$	ref. [72]	-1.251

Supplementary Table 2: Structure matching results for the test set when the space group is included in the prompt. The Reduced Unseen column represents the results for formulas that were not seen in training with any Z. (For example, if Na1Cl1 were in training, Na2Cl2 may be in All but not in Reduced Unseen.)

	All	Reduced Unseen
At least 1 match within 3 attempts	88.1%	86.3%
All 3 attempts matching	67.4%	70.0%
Matched on 1st attempt	78.4%	78.7%

Supplementary Table 3: Performance of the small model on the Challenge Set. E_f is the formation energy per atom. No space group was included in the prompt.

Composition	Mean E_f (eV/atom)	Min. E_f (eV/atom)	% Valid	Any match true?
$AlCu_2As(HO)_{12}$	-0.366	-0.568	19	no
Ba_2AuIO_6	-1.546	-1.599	96	no
$Ba_2Fe_2F_9$	-2.324	-2.598	14	no
$Ba_2Gd(BO_3)_2F$	-2.091	-2.578	12	no
Ba_2HfF_8	-3.427	-3.940	35	yes
Ba_2MnCr	0.985	0.611	100	yes
Ba_3GeTeS_4	-0.985	-1.366	33	no
$Ba_4GeSb_2Se_{11}$	-0.707	-0.859	18	no
$Ba_6Fe_2Te_3S_7$	-0.743	-0.989	15	no
$Ba_9Yb_2(SiO_4)_6$	-1.524	-1.524	1	no
$BaY_{16}Si_4O_{33}$	-	-	0	no
$CH_3NH_3PbI_3$	0.339	0.110	11	no
$Ca_{10}(PO_4)_6(OH)_2$	-	-	0	no
$Ca_2Bi_2O_7$	-1.759	-1.889	100	yes
$Ca_2Te_3O_8$	-	-	0	no
$CaFe_6Ge_6$	0.232	-0.207	26	yes
$CaHPO_3$	-1.471	-2.030	18	no
$CaPt_4P_6$	-0.330	-0.671	29	no
$CaZnV_2O_6$	-1.981	-2.551	32	yes
$Ce_6Cd_{23}Te$	-0.309	-0.369	91	yes
$Co_2CO_3(OH)_2$	-0.180	-0.527	29	no
$Cs_2Al_2O_3F_2$	-1.959	-2.455	16	no
$Cs_3LuSi_3O_9$	-	-	0	no
$Cs_8Cu_3Si_{14}O_{35}$	-	-	0	no
$CsCuTePt$	0.136	0.092	100	yes
$Cu_2C_1O_5H_2$	-0.279	-0.708	48	no
$Cu_3(CO_3)_2(OH)_2$	-0.164	-0.479	22	no
$Cu_4FeGe_2S_7$	-0.060	-0.266	39	no
$Eu_2FeGe_2OS_6$	-0.924	-1.265	45	yes
HgB_2S_4	0.336	0.051	18	no
$Ho_2Ir_3Si_5$	-0.883	-0.890	98	yes
K_2AgMoI_6	-0.638	-0.643	100	yes
$K_2Sr_4(PO_3)_{10}$	-	-	0	no
$K_6Zn(CO_3)_4$	-	-	0	no
$KScP_2O_7$	-2.626	-2.794	81	yes
$La_4Ga_2S_8O_3$	-1.024	-1.288	5	no
$LaScSe_3$	-1.879	-1.978	98	yes
Li_2GeS_3	-0.554	-0.960	44	no
$Li_9Al_4Sn_5$	-0.122	-0.189	2	no
$LiBa_2AlO_4$	-1.837	-2.053	3	no
$LiMnBi$	0.130	0.075	100	no
$LiTa_2NiSe_5$	-0.693	-0.847	71	no
$Mg_7Pt_4Ge_4$	-0.263	-0.521	23	no
MgF_2	-3.512	-3.811	93	yes
$Mn_4(PO_4)_3$	-1.750	-2.014	16	yes
$Na_2Hf(BO_3)_2$	-2.766	-2.835	69	yes
$Na_3Te_2(FeO_4)_3$	-1.362	-1.455	97	yes
$Na_4Sn_2Ge_5O_{16}$	-0.741	-0.867	2	no
$Na_5Mn_4P_4H_4(O_9F_2)_2$	-0.917	-0.948	2	no
$Na_6Li_4WO_4(CO_3)_4$	-	-	0	no
$NaGdSi_2O_6$	-2.721	-3.060	63	no
$NaMgV_5(H_5O_6)_4$	-	-	0	no
$NaSb_2TeO_7$	-0.855	-1.049	3	no
$NaSbSe_2O_7$	-0.528	-0.813	10	no
$Nd_3BSi_2O_{10}$	-	-	0	no
$Ni_3Te_2O_2(PO_4)_2(OH)_4$	-0.538	-0.824	28	no
$PbCu(OH)_2SO_4$	-0.362	-0.731	51	no
Rb_3SnCl_7	-1.329	-1.506	52	yes
$RbNiFe(PO_4)_2$	-0.896	-1.315	9	no
Sm_2BO_4	-2.978	-3.011	92	yes
$Sr(ClO_4)_2$	-0.044	-0.357	20	no
$Sr_2Bi_2O_7$	-1.729	-1.931	97	yes
$Sr_6Ge_3OSe_{11}$	-0.768	-1.017	4	no
$SrCo_4(OH)(PO_4)_3$	-0.868	-0.868	1	no
$Tb_3S_3BO_3$	-1.173	-2.026	45	no
Tb_3TeBO_9	-2.274	-2.477	73	yes
$YbMn_6Sn_6$	-0.042	-0.071	100	yes
$Zn_2(HTeO_3)(AsO_4)$	-0.556	-1.209	26	no
Zn_2BS_3Br	-0.077	-0.602	77	no
$Zn_4CuH_6(CO_6)_2$	-0.207	-0.641	19	no

Supplementary Table 4: Performance of the small model on the Challenge Set. E_f is the formation energy per atom. The space group was included in the prompt.

Composition	Mean E_f (eV/atom)	Min. E_f (eV/atom)	% Valid	Any match true?
$AlCu_2As(HO)_{12}$	-0.403	-0.735	50	no
Ba_2AuIO_6	-1.410	-1.608	74	yes
$Ba_2Fe_2F_9$	-2.163	-2.416	8	no
$Ba_2Gd(BO_3)_2F$	-1.869	-2.266	12	no
Ba_2HfF_8	-3.565	-4.082	40	yes
Ba_2MnCr	1.020	0.833	100	yes
Ba_3GeTeS_4	-1.148	-1.589	42	yes
$Ba_4GeSb_2Se_{11}$	-0.707	-0.888	16	no
$Ba_6Fe_2Te_3S_7$	-0.563	-1.020	6	no
$Ba_9Yb_2(SiO_4)_6$	-	-	0	no
$BaY_{16}Si_4O_{33}$	-	-	0	no
$CH_3NH_3PbI_3$	0.523	0.068	47	no
$Ca_{10}(PO_4)_6(OH)_2$	-	-	0	no
$Ca_2Bi_2O_7$	-1.758	-1.887	100	yes
$Ca_2Te_3O_8$	-	-	0	no
$CaFe_6Ge_6$	0.013	-0.175	8	yes
$CaHPO_3$	-1.175	-1.917	18	no
$CaPt_4P_6$	-0.346	-0.645	31	yes
$CaZnV_2O_6$	-2.197	-2.583	51	yes
$Ce_6Cd_{23}Te$	-0.315	-0.357	89	yes
$Co_2CO_3(OH)_2$	-0.086	-0.304	21	no
$Cs_2Al_2O_3F_2$	-2.028	-2.813	20	no
$Cs_3LuSi_3O_9$	-1.505	-1.927	3	no
$Cs_8Cu_3Si_{14}O_{35}$	-	-	0	no
$CsCuTePt$	0.136	0.128	100	yes
$Cu_2C_1O_5H_2$	-0.135	-0.541	29	no
$Cu_3(CO_3)_2(OH)_2$	-0.157	-0.581	29	no
$Cu_4FeGe_2S_7$	-0.055	-0.314	57	no
$Eu_2FeGe_2OS_6$	-1.151	-1.290	52	yes
HgB_2S_4	0.272	-0.043	25	no
$Ho_2Ir_3Si_5$	-0.881	-0.892	97	yes
K_2AgMoI_6	-0.638	-0.643	100	yes
$K_2Sr_4(PO_3)_{10}$	-	-	0	no
$K_6Zn(CO_3)_4$	-0.820	-0.820	1	no
$KScP_2O_7$	-2.703	-2.798	77	yes
$La_4Ga_2S_8O_3$	-1.028	-1.460	19	no
$LaScSe_3$	-1.912	-1.975	99	yes
Li_2GeS_3	-0.590	-0.962	24	yes
$Li_9Al_4Sn_5$	-0.117	-0.216	9	no
$LiBa_2AlO_4$	-1.392	-2.074	15	no
$LiMnBi$	0.123	-0.037	100	yes
$LiTa_2NiSe_5$	-0.249	-0.555	48	no
$Mg_7Pt_4Ge_4$	-0.412	-0.602	19	no
MgF_2	-3.774	-3.810	100	yes
$Mn_4(PO_4)_3$	-1.574	-1.997	34	yes
$Na_2Hf(BO_3)_2$	-2.802	-2.839	99	yes
$Na_3Te_2(FeO_4)_3$	-1.363	-1.458	94	yes
$Na_4Sn_2Ge_5O_{16}$	-0.974	-1.215	3	no
$Na_5Mn_4P_4H_4(O_9F_2)_2$	-1.053	-1.053	1	no
$Na_6Li_4WO_4(CO_3)_4$	-0.712	-1.012	3	no
$NaGdSi_2O_6$	-1.696	-2.620	12	no
$NaMgV_5(H_5O_6)_4$	-	-	0	no
$NaSb_2TeO_7$	-0.752	-0.752	1	no
$NaSbSe_2O_7$	-0.543	-1.081	21	no
$Nd_3BSi_2O_{10}$	-1.915	-1.915	1	no
$Ni_3Te_2O_2(PO_4)_2(OH)_4$	-0.582	-0.947	24	no
$PbCu(OH)_2SO_4$	-0.378	-0.776	46	no
Rb_3SnCl_7	-1.454	-1.538	76	yes
$RbNiFe(PO_4)_2$	-0.887	-1.082	4	no
Sm_2BO_4	-2.981	-3.011	92	yes
$Sr(ClO_4)_2$	-0.064	-0.158	2	no
$Sr_2Bi_2O_7$	-1.762	-1.968	100	yes
$Sr_6Ge_3OSe_{11}$	-0.672	-0.912	5	no
$SrCo_4(OH)(PO_4)_3$	-	-	0	no
$Tb_3S_3BO_3$	-1.281	-1.477	6	no
Tb_3TeBO_9	-2.251	-2.466	84	yes
$YbMn_6Sn_6$	-0.052	-0.066	99	yes
$Zn_2(HTeO_3)(AsO_4)$	-0.865	-1.210	50	no
Zn_2BS_3Br	0.111	-0.181	56	no
$Zn_4CuH_6(CO_6)_2$	-0.205	-0.302	4	no

Supplementary Table 5: Performance of the large model on the Challenge Set. E_f is the formation energy per atom. No space group was included in the prompt.

Composition	Mean E_f (eV/atom)	Min. E_f (eV/atom)	% Valid	Any match true?
$AlCu_2As(HO)_{12}$	-0.333	-0.620	33	no
Ba_2AuIO_6	-1.541	-1.589	65	no
$Ba_2Fe_2F_9$	-2.292	-2.701	10	no
$Ba_2Gd(BO_3)_2F$	-1.996	-2.207	5	no
Ba_2HfF_8	-3.522	-4.109	23	yes
Ba_2MnCr	1.027	0.593	100	yes
Ba_3GeTeS_4	-1.346	-1.681	71	yes
$Ba_4GeSb_2Se_{11}$	-1.085	-1.137	82	yes
$Ba_6Fe_2Te_3S_7$	-0.722	-1.036	12	no
$Ba_9Yb_2(SiO_4)_6$	-2.970	-3.244	41	yes
$BaY_{16}Si_4O_{33}$	-	-	0	no
$CH_3NH_3PbI_3$	-0.352	-0.358	100	yes
$Ca_{10}(PO_4)_6(OH)_2$	-2.999	-3.003	93	no
$Ca_2Bi_2O_7$	-1.751	-1.888	100	yes
$Ca_2Te_3O_8$	-	-	0	no
$CaFe_6Ge_6$	0.207	-0.003	7	no
$CaHPO_3$	-1.165	-1.504	14	no
$CaPt_4P_6$	-0.437	-0.756	34	yes
$CaZnV_2O_6$	-2.394	-2.585	64	yes
$Ce_6Cd_{23}Te$	-0.325	-0.342	93	yes
$Co_2CO_3(OH)_2$	-1.009	-1.019	100	yes
$Cs_2Al_2O_3F_2$	-2.114	-2.685	14	no
$Cs_3LuSi_3O_9$	-2.815	-2.946	19	no
$Cs_8Cu_3Si_{14}O_{35}$	-	-	0	no
$CsCuTePt$	0.140	0.131	100	yes
$Cu_2C_1O_5H_2$	-0.924	-0.933	99	yes
$Cu_3(CO_3)_2(OH)_2$	-1.007	-1.018	95	yes
$Cu_4FeGe_2S_7$	-0.073	-0.282	53	no
$Eu_2FeGe_2OS_6$	-0.824	-1.264	31	yes
HgB_2S_4	0.380	0.154	9	no
$Ho_2Ir_3Si_5$	-0.880	-0.887	99	yes
K_2AgMoI_6	-0.637	-0.644	100	yes
$K_2Sr_4(PO_3)_{10}$	-	-	0	no
$K_6Zn(CO_3)_4$	-	-	0	no
$KScP_2O_7$	-2.761	-2.795	100	yes
$La_4Ga_2S_8O_3$	-1.174	-1.421	5	no
$LaScSe_3$	-1.866	-1.940	96	yes
Li_2GeS_3	-0.735	-0.917	23	no
$Li_9Al_4Sn_5$	-0.112	-0.218	14	no
$LiBa_2AlO_4$	-2.000	-2.628	9	no
$LiMnBi$	0.124	-0.055	99	yes
$LiTa_2NiSe_5$	-0.436	-0.844	58	no
$Mg_7Pt_4Ge_4$	-	-	0	no
MgF_2	-3.380	-3.803	93	yes
$Mn_4(PO_4)_3$	-1.980	-2.020	81	no
$Na_2Hf(BO_3)_2$	-2.567	-2.829	75	yes
$Na_3Te_2(FeO_4)_3$	-1.430	-1.460	100	yes
$Na_4Sn_2Ge_5O_{16}$	-1.023	-1.034	3	no
$Na_5Mn_4P_4H_4(O_9F_2)_2$	-1.094	-1.151	2	no
$Na_6Li_4WO_4(CO_3)_4$	-	-	0	no
$NaGdSi_2O_6$	-2.962	-3.083	73	yes
$NaMgV_5(H_5O_6)_4$	-	-	0	no
$NaSb_2TeO_7$	-0.689	-0.943	4	no
$NaSbSe_2O_7$	-0.497	-0.698	9	no
$Nd_3BSi_2O_{10}$	-3.338	-3.374	86	yes
$Ni_3Te_2O_2(PO_4)_2(OH)_4$	-0.502	-1.010	31	no
$PbCu(OH)_2SO_4$	-1.153	-1.160	98	yes
Rb_3SnCl_7	-1.438	-1.481	6	yes
$RbNiFe(PO_4)_2$	-1.011	-1.557	9	no
Sm_2BO_4	-2.986	-3.006	95	yes
$Sr(ClO_4)_2$	-0.170	-0.348	12	yes
$Sr_2Bi_2O_7$	-1.672	-1.874	100	yes
$Sr_6Ge_3OSe_{11}$	-0.870	-0.870	1	no
$SrCo_4(OH)(PO_4)_3$	-	-	0	no
$Tb_3S_3BO_3$	-1.792	-2.195	13	no
Tb_3TeBO_9	-1.994	-2.347	71	yes
$YbMn_6Sn_6$	-0.056	-0.067	100	yes
$Zn_2(HTeO_3)(AsO_4)$	-0.470	-0.671	22	no
Zn_2BS_3Br	0.024	-0.510	56	no
$Zn_4CuH_6(CO_6)_2$	-0.181	-0.550	18	no

Supplementary Table 6: Performance of the large model on the Challenge Set. E_f is the formation energy per atom. The space group was included in the prompt.

Composition	Mean E_f (eV/atom)	Min. E_f (eV/atom)	% Valid	Any match true?
$AlCu_2As(HO)_{12}$	-0.343	-0.540	30	no
Ba_2AuIO_6	-1.411	-1.572	65	yes
$Ba_2Fe_2F_9$	-2.175	-2.503	4	no
$Ba_2Gd(BO_3)_2F$	-2.505	-2.739	4	no
Ba_2HfF_8	-3.466	-4.058	47	yes
Ba_2MnCr	1.066	0.862	100	yes
Ba_3GeTeS_4	-1.520	-1.681	70	yes
$Ba_4GeSb_2Se_{11}$	-1.100	-1.138	75	yes
$Ba_6Fe_2Te_3S_7$	-0.724	-1.068	31	no
$Ba_9Yb_2(SiO_4)_6$	-2.949	-3.241	38	yes
$BaY_{16}Si_4O_{33}$	-	-	0	no
$CH_3NH_3PbI_3$	-0.358	-0.358	100	yes
$Ca_{10}(PO_4)_6(OH)_2$	-1.634	-1.634	1	no
$Ca_2Bi_2O_7$	-1.745	-1.864	100	yes
$Ca_2Te_3O_8$	-	-	0	no
$CaFe_6Ge_6$	0.079	-0.063	12	no
$CaHPO_3$	-1.159	-1.857	22	no
$CaPt_4P_6$	-0.475	-0.813	33	yes
$CaZnV_2O_6$	-2.310	-2.571	79	yes
$Ce_6Cd_{23}Te$	-0.327	-0.360	99	yes
$Co_2CO_3(OH)_2$	-1.009	-1.020	100	yes
$Cs_2Al_2O_3F_2$	-2.190	-2.831	18	no
$Cs_3LuSi_3O_9$	-	-	0	no
$Cs_8Cu_3Si_{14}O_{35}$	-	-	0	no
$CsCuTePt$	0.139	0.131	100	yes
$Cu_2C_1O_5H_2$	-0.923	-0.929	99	yes
$Cu_3(CO_3)_2(OH)_2$	-0.260	-0.634	27	no
$Cu_4FeGe_2S_7$	-0.104	-0.215	60	no
$Eu_2FeGe_2OS_6$	-1.123	-1.300	91	yes
HgB_2S_4	0.191	0.040	10	no
$Ho_2Ir_3Si_5$	-0.881	-0.886	99	yes
K_2AgMoI_6	-0.637	-0.643	100	yes
$K_2Sr_4(PO_3)_{10}$	-	-	0	no
$K_6Zn(CO_3)_4$	-0.857	-0.997	2	no
$KScP_2O_7$	-2.759	-2.803	98	yes
$La_4Ga_2S_8O_3$	-1.235	-1.290	4	no
$LaScSe_3$	-1.880	-1.960	97	yes
Li_2GeS_3	-0.629	-0.930	15	yes
$Li_9Al_4Sn_5$	-0.149	-0.249	37	no
$LiBa_2AlO_4$	-1.621	-2.328	37	no
$LiMnBi$	0.066	0.001	100	yes
$LiTa_2NiSe_5$	-0.166	-0.493	50	no
$Mg_7Pt_4Ge_4$	-0.448	-0.591	50	no
MgF_2	-3.783	-3.808	100	yes
$Mn_4(PO_4)_3$	-1.903	-2.021	84	yes
$Na_2Hf(BO_3)_2$	-2.802	-2.857	98	yes
$Na_3Te_2(FeO_4)_3$	-1.430	-1.456	100	yes
$Na_4Sn_2Ge_5O_{16}$	-0.946	-1.214	15	no
$Na_5Mn_4P_4H_4(O_9F_2)_2$	-0.902	-1.123	2	no
$Na_6Li_4WO_4(CO_3)_4$	-0.914	-1.307	3	no
$NaGdSi_2O_6$	-3.011	-3.083	79	yes
$NaMgV_5(H_5O_6)_4$	-	-	0	no
$NaSb_2TeO_7$	-0.717	-0.892	4	no
$NaSbSe_2O_7$	-0.575	-0.983	21	no
$Nd_3BSi_2O_{10}$	-3.363	-3.372	76	yes
$Ni_3Te_2O_2(PO_4)_2(OH)_4$	-0.588	-0.851	21	no
$PbCu(OH)_2SO_4$	-1.138	-1.161	99	yes
Rb_3SnCl_7	-1.431	-1.521	51	yes
$RbNiFe(PO_4)_2$	-0.966	-1.218	6	no
Sm_2BO_4	-2.986	-3.005	96	yes
$Sr(ClO_4)_2$	-0.184	-0.337	11	yes
$Sr_2Bi_2O_7$	-1.671	-1.861	100	yes
$Sr_6Ge_3OSe_{11}$	-0.698	-0.836	6	no
$SrCo_4(OH)(PO_4)_3$	-0.506	-0.506	1	no
$Tb_3S_3BO_3$	-1.246	-1.410	7	no
Tb_3TeBO_9	-2.035	-2.443	78	yes
$YbMn_6Sn_6$	-0.055	-0.070	100	yes
$Zn_2(HTeO_3)(AsO_4)$	-0.389	-0.889	34	no
Zn_2BS_3Br	0.167	-0.245	21	no
$Zn_4CuH_6(CO_6)_2$	-0.065	-0.126	3	no

Supplementary Table 7: Metrics for the unconditional generation tasks.* The CDVAE results are from Xie et al. [13]. The DiffCSP and DiffCSP++ results are from Jiao et al. [73, 74]. The UniMat results are from Yang et al. [75]. The LM-CH (character-level tokenization) and LM-AC (atom+coordinate-level tokenization) results are from Flam-Shepherd et al. [76]. The LLaMA 70B results are from Gruver et al. [77]. Abbreviations and symbols: τ: sampling temperature; d_ρ and d_{elem}: Wasserstein distances between the generated and testing structures, in terms of density and the number of elements, respectively; COV: coverage; AMSD: average minimum structure distance; AMCD: average minimum composition distance; -R: recall; -P: precision.

	Validity (%) ↑		Coverage (%) ↑		Property ↓		Average Minimum Distance ↓			
Method	Struct	Comp	COV-R	COV-P	d_ρ	d_{elem}	AMSD-R	AMSD-P	AMCD-R	AMCD-P
					MP-20					
CDVAE	**100.0**	86.70	99.15	99.49	0.6875	1.4320	0.154	0.188	3.620	4.014
DiffCSP	**100.0**	83.25	99.71	**99.76**	0.3502	0.3398	-	-	-	-
DiffCSP++	99.94	85.12	99.73	99.59	0.2351	0.3749	-	-	-	-
UnitMat	97.20	89.40	**99.80**	99.70	**0.0880**	**0.0560**	0.097	0.119	**2.410**	2.410
LM-CH	84.81	83.55	99.25	97.89	0.8640	0.1320	-	-	-	-
LM-AC	95.81	88.87	99.60	98.55	0.6960	0.0920	-	-	-	-
LLaMA 70B (τ=1.0)	96.50	86.30	96.80	98.30	1.7200	0.5500	-	-	-	-
LLaMA 70B (τ=0.7)	99.60	**95.40**	85.80	98.90	0.8100	0.4400	-	-	-	-
CrystaLLM small (τ=0.7)	93.66	91.10	98.52	95.08	0.8353	0.2229	0.096	0.096	3.251	2.084
CrystaLLM small (τ=0.5)	94.97	93.80	97.58	95.75	1.1824	0.3269	0.106	0.095	3.729	**1.762**
CrystaLLM large (τ=0.7)	95.54	93.07	97.22	96.40	0.5965	0.1709	**0.090**	0.077	3.299	2.114
CrystaLLM large (τ=0.5)	96.21	**95.40**	96.78	96.60	0.9835	0.3436	0.098	**0.076**	3.675	1.880
					Perov-5					
CDVAE	**100.0**	98.59	99.45	98.46	0.1258	0.0628	0.048	0.059	**0.696**	**1.270**
DiffCSP	**100.0**	98.85	**99.74**	98.27	0.1110	0.0128	-	-	-	-
DiffCSP++	**100.0**	98.77	99.60	98.80	**0.0661**	**0.0040**	-	-	-	-
UnitMat	**100.0**	98.80	99.20	98.20	0.0760	0.0250	0.046	0.074	0.711	1.399
LM-CH	**100.0**	98.51	99.60	**99.42**	0.0710	0.0360	-	-	-	-
LM-AC	**100.0**	98.79	98.78	99.36	0.0890	0.0280	-	-	-	-
CrystaLLM small (τ=0.7)	99.90	99.04	98.20	99.01	0.3355	0.0299	**0.025**	0.027	1.055	1.287
CrystaLLM small (τ=0.5)	99.83	**99.24**	97.91	98.95	0.3950	0.0970	0.027	0.025	1.215	1.293
CrystaLLM large (τ=0.7)	99.82	98.92	98.28	98.92	0.2070	0.0490	0.026	0.024	1.000	1.288
CrystaLLM large (τ=0.5)	99.96	98.86	97.86	98.73	0.3937	0.1240	0.027	**0.020**	1.144	1.319
					Carbon-24					
CDVAE	**100.0**	-	99.80	83.08	0.1407	-	0.048	0.134	0.000	0.000
DiffCSP	**100.0**	-	99.90	97.27	0.0805	-	-	-	-	-
DiffCSP++	99.99	-	**100.0**	88.28	0.0307	-	-	-	-	-
UnitMat	**100.0**	-	**100.0**	96.50	**0.0130**	-	0.018	0.052	0.000	0.000
CrystaLLM small (τ=0.7)	99.21	-	99.85	97.03	0.0639	-	0.015	0.021	0.000	0.000
CrystaLLM small (τ=0.5)	99.86	-	99.80	98.96	0.1217	-	0.022	0.012	0.000	0.000
CrystaLLM large (τ=0.7)	99.70	-	99.80	98.37	0.0409	-	**0.014**	0.018	0.000	0.000
CrystaLLM large (τ=0.5)	99.90	-	99.75	**99.52**	0.0953	-	0.018	**0.010**	0.000	0.000

* Numbers in bold indicate the best results for the given task.

Supplementary Table 8: Monte Carlo Tree Search (MCTS) results for the small model. E_f is the formation energy per atom. No space group was included in the prompt.

Composition	Algorithm	Best E_f (eV/atom)	Best Iter.	Mean E_f (eV/atom)	% Valid
$Ba_2Fe_2F_9$	Random	-2.787	10	-2.273	14.50
	MCTS	-2.812	570	-2.359	42.10
$Ba_2Gd(BO_3)_2F$	Random	-2.950	943	-2.121	18.50
	MCTS	-2.992	699	-2.104	27.20
$Ba_4GeSb_2Se_{11}$	Random	-0.928	571	-0.704	16.70
	MCTS	-0.925	509	-0.735	37.40
$Ba_6Fe_2Te_3S_7$	Random	-1.123	110	-0.689	16.60
	MCTS	-1.216	510	-0.730	17.40
$Ba_9Yb_2(SiO_4)_6$	Random	-2.712	834	-1.907	1.40
	MCTS	-2.777	534	-1.815	2.10
$CH_3NH_3PbI_3$	Random	-0.027	257	0.431	10.60
	MCTS	-0.199	611	0.448	52.80
$CaHPO_3$	Random	-2.048	55	-1.397	18.10
	MCTS	-2.247	367	-1.596	59.30
$Cs_2Al_2O_3F_2$	Random	-2.825	283	-1.972	22.90
	MCTS	-2.922	651	-2.051	37.90
HgB_2S_4	Random	-0.142	765	0.284	16.90
	MCTS	-0.212	282	0.270	34.20
$La_4Ga_2S_8O_3$	Random	-1.404	696	-1.016	3.30
	MCTS	-1.495	27	-1.094	5.70
$Li_9Al_4Sn_5$	Random	-0.225	409	-0.147	1.50
	MCTS	-0.231	123	-0.143	4.60
$LiBa_2AlO_4$	Random	-2.683	667	-1.728	4.40
	MCTS	-2.504	281	-1.854	61.20
$Mn_4(PO_4)_3$	Random	-2.029	122	-1.787	22.00
	MCTS	-2.045	632	-1.946	68.90
$Na_4Sn_2Ge_5O_{16}$	Random	-1.126	40	-0.863	2.50
	MCTS	-1.264	848	-0.915	8.70
$Na_5Mn_4P_4H_4(O_9F_2)_2$	Random	-1.510	660	-1.020	2.40
	MCTS	-1.531	335	-0.979	1.10
$NaSb_2TeO_7$	Random	-1.292	64	-0.851	4.50
	MCTS	-1.391	787	-0.875	25.50
$NaSbSe_2O_7$	Random	-0.969	795	-0.473	11.50
	MCTS	-1.108	792	-0.658	42.70
$RbNiFe(PO_4)_2$	Random	-1.465	197	-0.835	4.70
	MCTS	-1.599	699	-1.044	6.80
$Sr_6Ge_3OSe_{11}$	Random	-0.974	658	-0.716	1.50
	MCTS	-1.214	207	-0.910	31.10
$SrCo_4(OH)(PO_4)_3$	Random	-1.245	493	-0.845	2.20
	MCTS	-1.223	102	-0.674	9.90

Supplementary Table 9: MCTS results for the small model. E_f is the formation energy per atom. The space group was included in the prompt.

Composition	Algorithm	Best E_f (eV/atom)	Best Iter.	Mean E_f (eV/atom)	% Valid
$Ba_2Fe_2F_9$	Random	-2.523	822	-2.213	8.10
	MCTS	-2.642	188	-2.204	8.30
$Ba_2Gd(BO_3)_2F$	Random	-2.761	906	-1.922	7.60
	MCTS	-2.644	461	-1.956	10.20
$Ba_4GeSb_2Se_{11}$	Random	-0.904	490	-0.677	15.30
	MCTS	-0.923	450	-0.725	25.30
$Ba_6Fe_2Te_3S_7$	Random	-1.110	297	-0.632	6.20
	MCTS	-1.137	160	-0.711	24.60
$CaFe_6Ge_6$	Random	-0.205	353	0.018	9.30
	MCTS	-0.209	675	0.065	8.70
$Cs_3LuSi_3O_9$	Random	-1.967	640	-1.491	1.60
	MCTS	-1.953	325	-1.514	1.40
$K_6Zn(CO_3)_4$	Random	-1.308	143	-0.694	1.50
	MCTS	-0.958	487	-0.673	1.40
$Li_9Al_4Sn_5$	Random	-0.247	926	-0.115	10.50
	MCTS	-0.290	559	-0.134	42.50
$LiBa_2AlO_4$	Random	-2.395	365	-1.370	15.60
	MCTS	-2.380	125	-1.319	16.40
$Na_4Sn_2Ge_5O_{16}$	Random	-1.271	849	-0.907	5.80
	MCTS	-1.432	119	-0.920	10.50
$Na_5Mn_4P_4H_4(O_9F_2)_2$	Random	-1.567	463	-1.105	2.10
	MCTS	-1.472	671	-1.076	3.50
$Na_6Li_4WO_4(CO_3)_4$	Random	-1.488	675	-0.677	6.80
	MCTS	-1.203	694	-0.722	2.90
$NaGdSi_2O_6$	Random	-2.486	128	-1.686	12.80
	MCTS	-2.591	893	-1.669	9.60
$NaSb_2TeO_7$	Random	-0.902	616	-0.571	1.40
	MCTS	-1.025	881	-0.667	5.40
$Nd_3BSi_2O_{10}$	Random	-2.910	740	-2.234	1.60
	MCTS	-2.777	411	-2.372	0.50
$RbNiFe(PO_4)_2$	Random	-1.477	104	-0.926	4.70
	MCTS	-1.555	73	-0.961	8.50
$Sr(ClO_4)_2$	Random	-0.278	125	-0.043	2.80
	MCTS	-0.314	633	-0.051	3.10
$Sr_6Ge_3OSe_{11}$	Random	-1.155	679	-0.763	3.80
	MCTS	-1.358	158	-0.874	3.00
$Tb_3S_3BO_3$	Random	-1.877	487	-1.358	6.10
	MCTS	-1.915	199	-1.327	5.70
$Zn_4CuH_6(CO_6)_2$	Random	-0.350	834	-0.139	3.70
	MCTS	-0.575	815	-0.255	4.00

Supplementary Table 10: Novel materials generated unconditionally with the large model. Only those with a DFT energy above the hull, E_{hull}, of less than or equal to 0.1 eV/atom are listed.

Composition	Z	Space Group	E_{hull} (eV/atom)
Ca_2YSbO_6	2	$P2_1/c$	0.00
$NaAlS_2$	16	$P2_1$	0.00
$Ba_4Na_2Ir_2O_{11}$	2	Cm	0.00
Li_2FeSiO_4	4	$Pna2_1$	0.02
La_2Al_{17}	3	$R\bar{3}m$	0.03
$Ba_4Zr_2Mo_2O_{11}$	2	Pm	0.03
$LaSc(Al_2Pd)_2$	2	Amm2	0.04
$KLi(NbCl_3)_6$	2	P1	0.05
$Li_4Mn_3Nb_2Fe_3O_{16}$	1	P1	0.05
Ba_2SrCa	2	Imm2	0.06
MnAlTcIr	4	$F\bar{4}3m$	0.07
$Na_5(SnS_4)_2$	4	$P2_1$	0.07
$MnVO_4$	4	Pbcn	0.07
$Li_4Ti_3V_3(SbO_8)_2$	2	Cm	0.07
$Li_4Ti_3Cr_3(FeO_8)_2$	2	Pc	0.07
$K_2LiV_5H_{10}O_{19}$	2	$P\bar{1}$	0.07
$KNaS_2$	2	$P\bar{1}$	0.09
Li_2CrCuH_6	4	$P2_1/c$	0.09
$Li_4Ti_3Fe_3(WO_8)_2$	2	P1	0.10
$MnAl_2V_3$	1	P4mm	0.10

Supplementary Figures

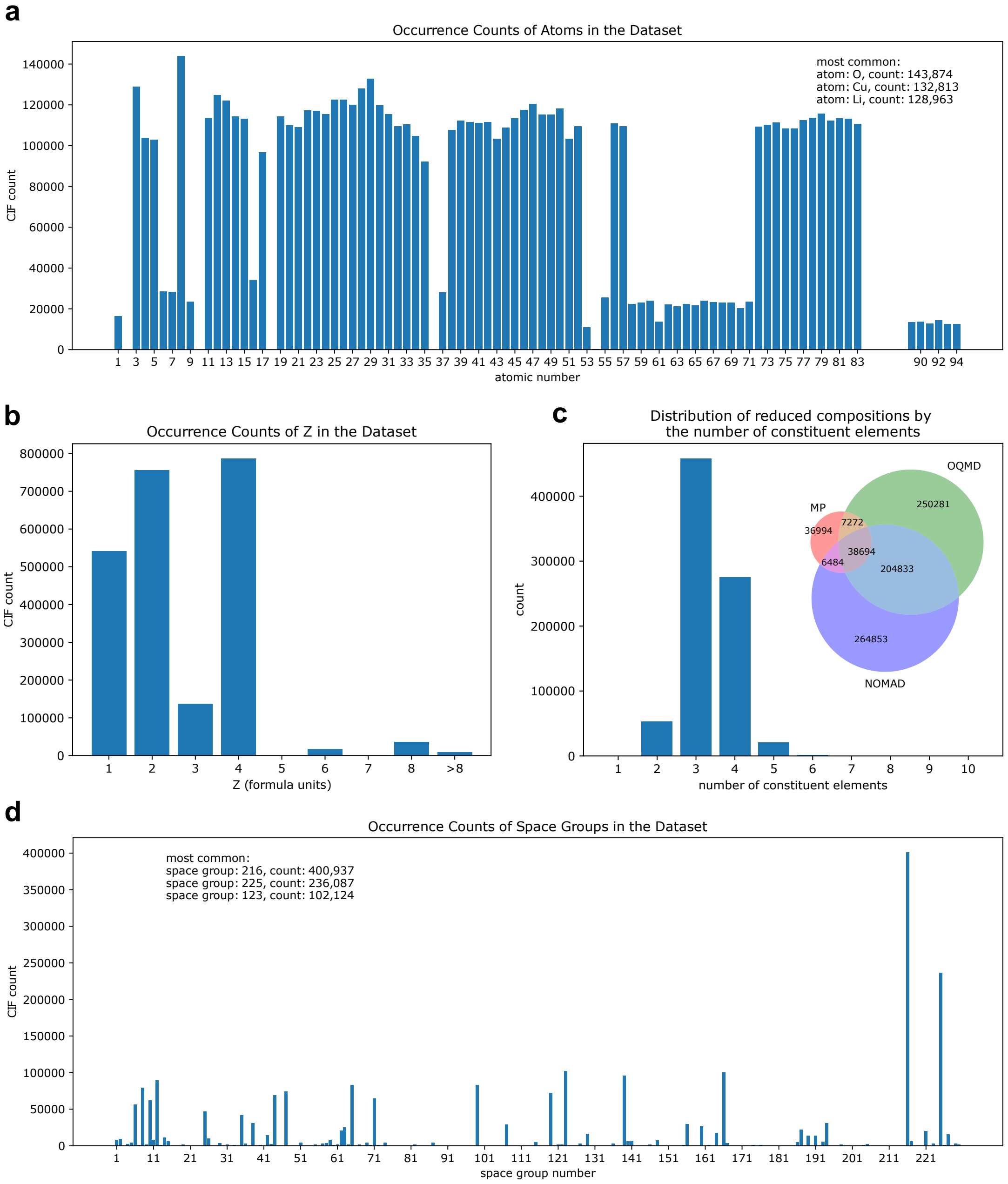

Supplementary Figure 1: Various plots describing the contents of the CIF file dataset. **a** The distribution of CIF files containing the atoms indicated (by atomic number) on the x-axis. The most abundant element in the dataset is oxygen, followed by copper, then lithium. **b** The distribution of Z values (i.e. the number of formula units in the unit cell) in the dataset. The majority of structures have Z of 1-4. **c** The distribution of compositions by the number of constituent elements in the formula. Most formulas are ternary or quaternary. Inset: The Venn diagram illustrates the numbers of unique reduced compositions obtained from each of the publicly accessible materials databases used to create the training dataset. **d** The distribution of space groups occurring in the CIF files of the dataset.

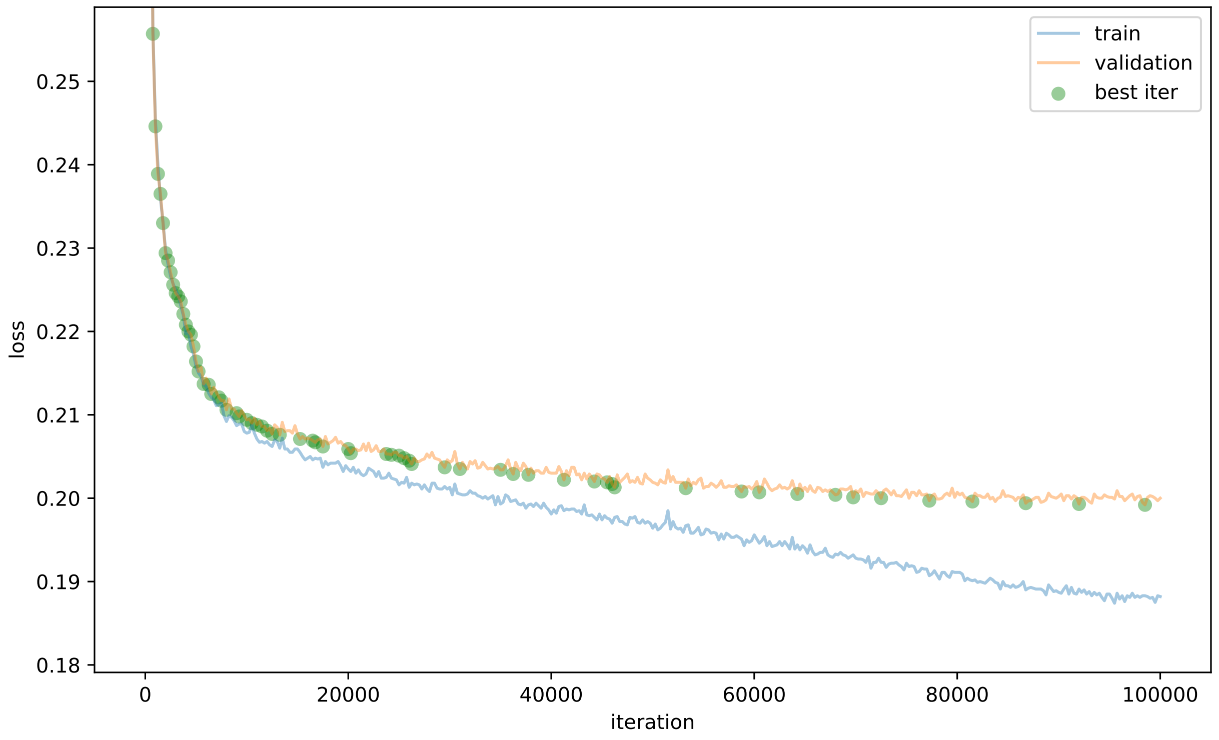

Supplementary Figure 2: A plot of the training set and validation set losses for the small model over the course of training. The green points represent an improved loss on the validation set, demonstrating that the model continues to improve its performance on the validation set even after 90,000 iterations. While the gap between the training set loss and the validation set loss appears to grow over the course of training, this is not necessarily indicative of overfitting. The growing gap could be more indicative of the differences between the distributions of the training and validation sets. On absolute terms, the difference between the curves is less than 0.02 units. Moreover, the performance on the validation and challenge sets indicate that the model trained for more iterations is superior.

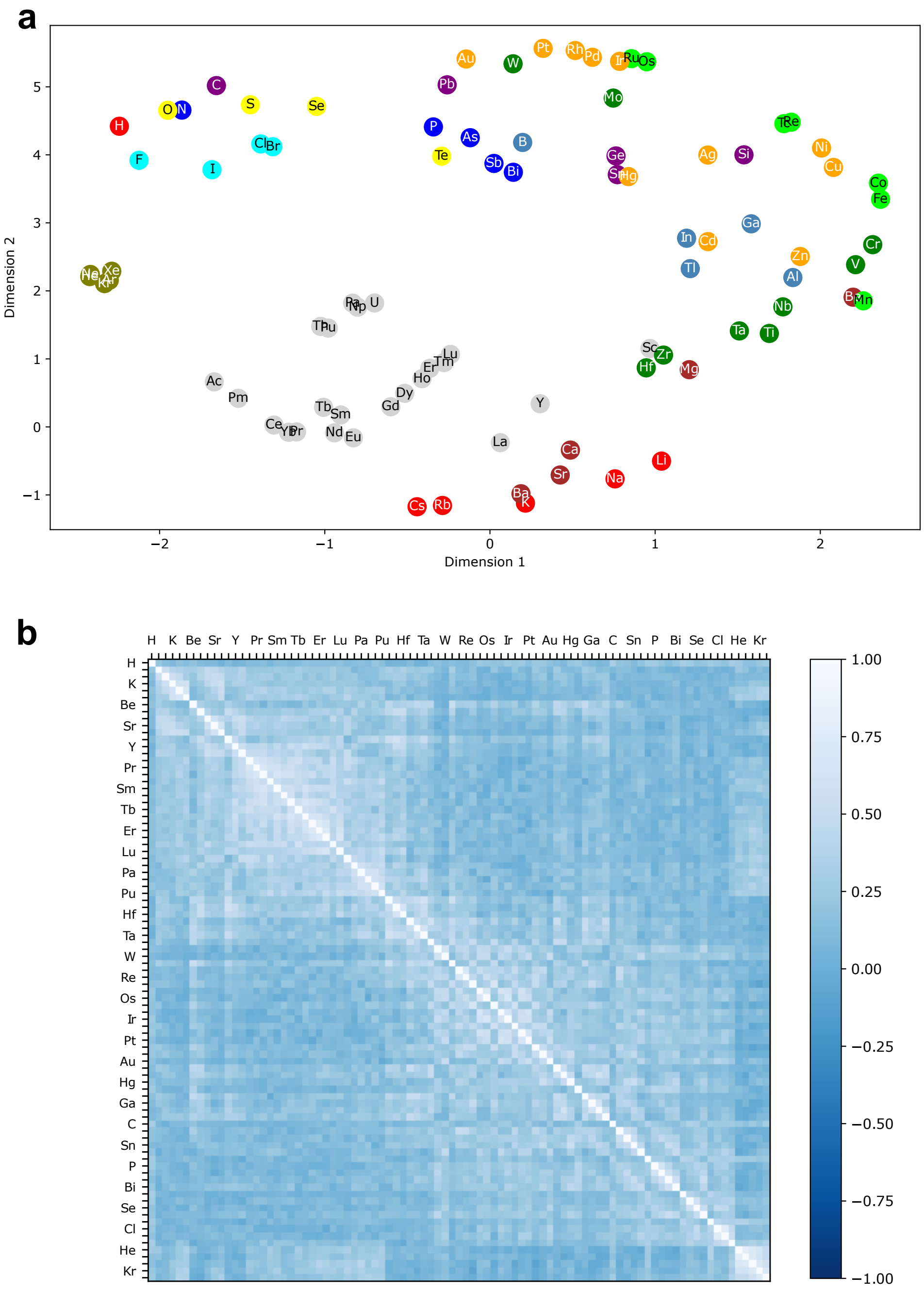

Supplementary Figure 3: Plots depicting the small model's learned atom vectors. **a** A t-SNE [78] plot of the small model's dimensionally reduced learned atom vectors. **b** A heatmap of the cosine similarities between the small model's learned atom vectors.

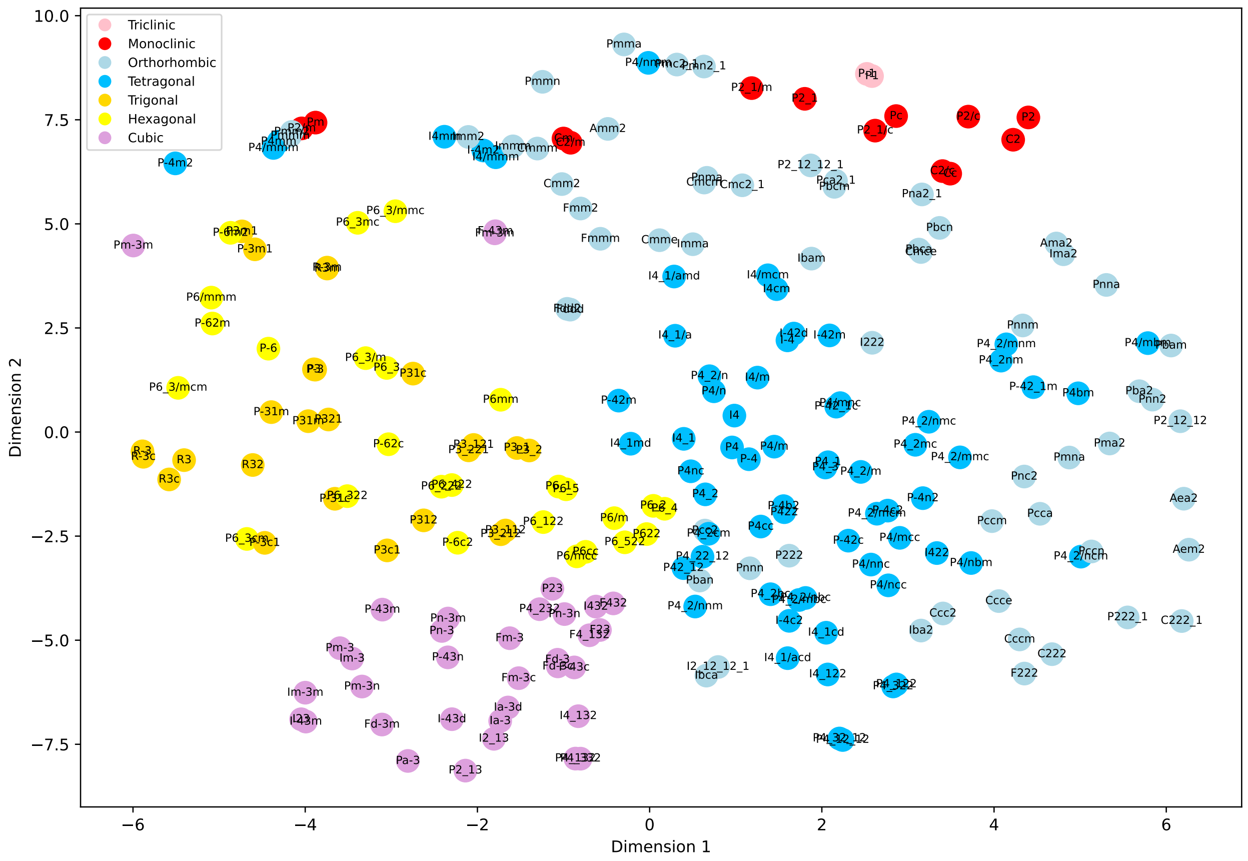

Supplementary Figure 4: A plot of the small model's learned space group vectors. The space group vectors were reduced to 2 dimensions using the t-SNE algorithm.

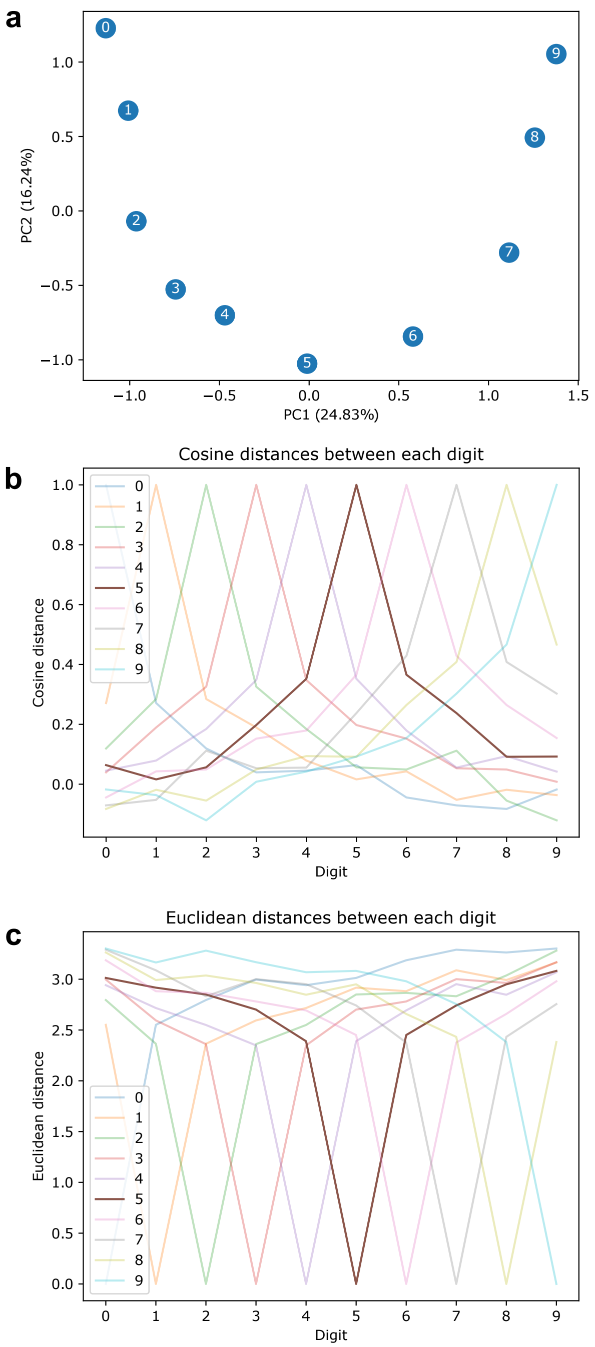

Supplementary Figure 5: Plots depicting the small model's learned numeric digit vectors. **a** A plot of the small model's learned numeric digit vectors, dimensionally reduced using PCA. **b** A plot of the cosine similarities between the small model's learned numeric digit vectors. **c** A plot of the Euclidean distances between the small model's learned numeric digit vectors.

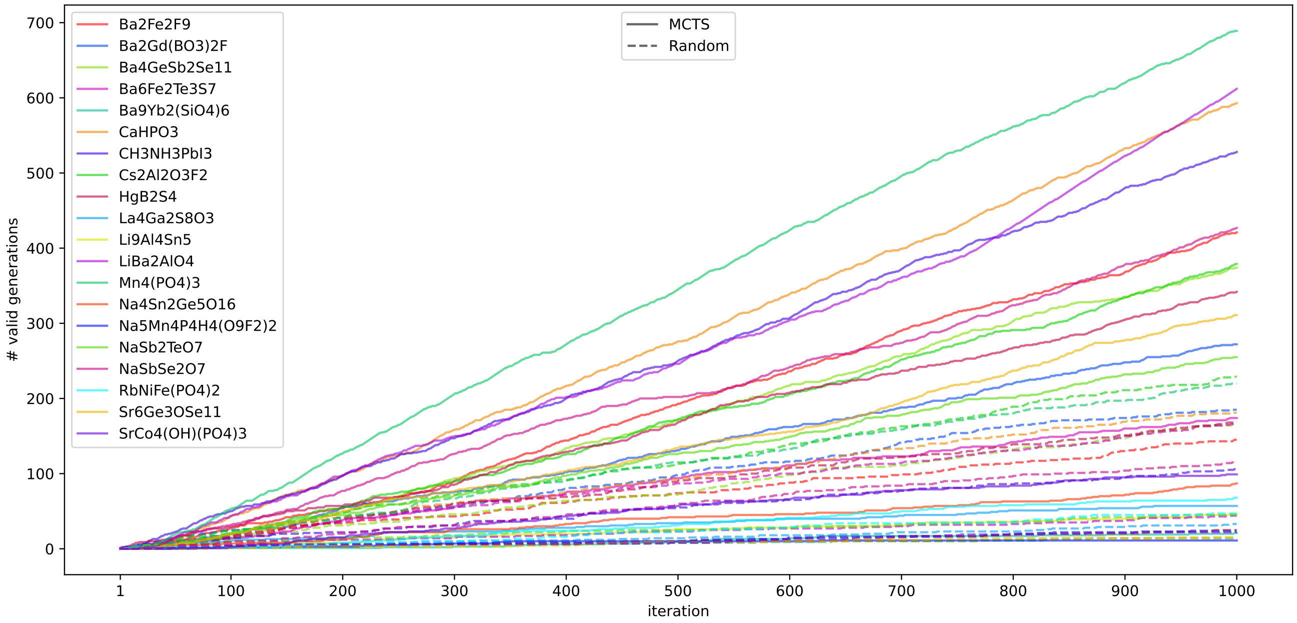

Supplementary Figure 6: Plots of the number of valid generations over the course of 1,000 iterations for the MCTS experiment (with no space group). The plot illustrates the finding that MCTS produces more valid generations than sampling randomly, and that, in some cases, the validation rate increases over time.

Supplementary References

[1] Urusov, V. & Nadezhina, T. Frequency distribution and selection of space groups in inorganic crystal chemistry. *Journal of Structural Chemistry* **50**, 22–37 (2009).

[2] Karpathy, A. nanoGPT: The simplest, fastest repository for training/finetuning medium-sized GPTs. `https://github.com/karpathy/nanoGPT` (2023).

[3] Radford, A., Narasimhan, K., Salimans, T., Sutskever, I. *et al.* Improving Language Understanding by Generative Pre-Training. Tech. Rep., OpenAI (2018). URL `https://cdn.openai.com/research-covers/language-unsupervised/language_understanding_paper.pdf`.

[4] Hendrycks, D. & Gimpel, K. Gaussian Error Linear Units (GELUs). *arXiv preprint arXiv:1606.08415* (2016).

[5] Press, O. & Wolf, L. Using the Output Embedding to Improve Language Models. *arXiv preprint arXiv:1608.05859* (2016).

[6] Loshchilov, I. & Hutter, F. Decoupled Weight Decay Regularization. *arXiv preprint arXiv:1711.05101* (2017).

[7] Togo, A. & Tanaka, I. Spglib: a software library for crystal symmetry search. *arXiv preprint arXiv:1808.01590* (2018).

[8] Coulom, R. Efficient Selectivity and Backup Operators in Monte-Carlo Tree Search. In *International Conference on Computers and Games*, 72–83 (Springer, 2006).

[9] Browne, C. B. *et al.* A Survey of Monte Carlo Tree Search Methods. *IEEE Transactions on Computational Intelligence and AI in games* **4**, 1–43 (2012).

[10] Rosin, C. D. Multi-armed Bandits with Episode Context. *Annals of Mathematics and Artificial Intelligence* **61**, 203–230 (2011).

[11] Silver, D. *et al.* Mastering the game of Go with deep neural networks and tree search. *Nature* **529**, 484–489 (2016).

[12] Choudhary, K. & DeCost, B. Atomistic Line Graph Neural Network for improved materials property predictions. *npj Computational Materials* **7**, 185 (2021).

[13] Xie, T., Fu, X., Ganea, O.-E., Barzilay, R. & Jaakkola, T. Crystal Diffusion Variational Autoencoder for Periodic Material Generation. *arXiv preprint arXiv:2110.06197* (2021).

[14] Zimmermann, N. E. & Jain, A. Local structure order parameters and site fingerprints for quantification of coordination environment and crystal structure similarity. *RSC advances* **10**, 6063–6081 (2020).

[15] Ward, L., Agrawal, A., Choudhary, A. & Wolverton, C. A general-purpose machine learning framework for predicting properties of inorganic materials. *npj Computational Materials* **2**, 1–7 (2016).

[16] Ganea, O. *et al.* GeoMol: Torsional Geometric Generation of Molecular 3D Conformer Ensembles. *Advances in Neural Information Processing Systems* **34**, 13757–13769 (2021).

[17] Plumhoff, A. *Thermodynamic properties, crystal structures, phase relations and isotopic studies of selected copper oxysalts.* Ph.D. thesis (2020). Friedrich-Schiller-Universität Jena.

[18] Pogue, E. A. *et al.* A gold (I) oxide double perovskite: Ba_2AuIO_6. *Journal of the American Chemical Society* **143**, 19033–19042 (2021).

[19] Huang, Q. *et al.* Investigation of Charge-Ordered Barium Iron Fluorides with One-Dimensional Structural Diversity and Complex Magnetic Interactions. *Inorganic Chemistry* **62**, 14044–14054 (2023).

[20] Frøen, E. H., Adler, P. & Valldor, M. Synthesis and Properties of $Ba_6Fe_2Te_3S_7$, with an Fe Dimer in a Magnetic Singlet State. *Inorganic Chemistry* **62**, 12548–12556 (2023).

[21] Chen, Y. *et al.* Improvement on Magnetocaloric Effect through Structural Evolution in Gadolinium Borate Halides $Ba_2Gd(BO_3)_2X$ (X= F, Cl). *Inorganic Chemistry* **62**, 15584–15592 (2023).

[22] Yuan, F.-Y. *et al.* $Ba_4GeSb_2Se_{11}$: an infrared nonlinear optical crystal with a V-shaped Se_{32}–group possessing a large contribution to the SHG response. *Inorganic Chemistry* **60**, 15593–15598 (2021).

[23] Yadav, S., Panigrahi, G., Niranjan, M. K. & Prakash, J. Ba_3GeTeS_4: A new quaternary heteroanionic chalcogenide semiconductor. *Journal of Solid State Chemistry* **323**, 124028 (2023).

[24] Keerthisinghe, N., Ayer, G. B., Smith, M. D. & Zur Loye, H.-C. Comparative Study on Crystal Structures and Synthetic Techniques of Ternary Hafnium/Zirconium Fluorides. *Inorganic Chemistry* **62**, 12089–12098 (2023).

[25] Motozawa, S., Kimura, H., Takahashi, J., Simura, R. & Yamane, H. $BaY_{16}Si_4O_{33}$ containing $Ba(SiO_4)_4$ orthosilicates. *Acta Crystallographica Section E: Crystallographic Communications* **78**, 1249–1252 (2022).

[26] Liu, A. *et al.* Ba_9RE_2 (SiO_4) 6 (RE= Ho–Yb): A Family of Rare-Earth-Based Honeycomb-Lattice Magnets. *Inorganic Chemistry* **62**, 13867–13876 (2023).

[27] Saiduzzaman, M. *et al.* Hydrothermal synthesis of pyrochlore-type pentavalent bismuthates $Ca_2Bi_2O_7$ and $Sr_2Bi_2O_7$. *Inorganic Chemistry* **58**, 1759–1763 (2019).

[28] Braun, T. & Hlukhyy, V. Structural order-disorder in $CaFe_6Ge_6$ and $Ca_1{-}xCo_6Ge_6$. *Journal of Solid State Chemistry* **318**, 123742 (2023).

[29] Phillips, M. L. & Harrison, W. T. Synthesis and crystal structure of calcium hydrogen phosphite, $CaHPO_3$. *Acta Crystallographica Section E: Crystallographic Communications* **75**, 997–1000 (2019).

[30] Makhaneva, A. Y., Zakharova, E. Y., Nesterenko, S. N., Lyssenko, K. A. & Kuznetsov, A. N. $CaPt_4P_6$, first calcium-containing representative of the ternary pyrite-derived pnictides of the $BaPt_4As_6$ type: Synthesis, crystal, and electronic structure. *Journal of Solid State Chemistry* **322**, 123969 (2023).

[31] Weil, M. $Ca_2Te_3O_8$, a new phase in the CaO–TeO_2 system. *Acta Crystallographica Section E: Crystallographic Communications* **75**, 26–29 (2019).

[32] Fukuda, M. *et al.* A-Site Columnar-Ordered Perovskite $CaZnV_2O_6$ as a Pauli-Paramagnetic Metal. *Inorganic Chemistry* (2023).

[33] Desroches, G. & Bobev, S. Synthesis and structure determination of $Ce_6Cd_{23}Te$: a new chalcogen-containing member of the $RE_6Cd_{23}T$ family (RE is a rare-earth metal and T is a late group 14, 15 and 16 element). *Acta Crystallographica Section C: Structural Chemistry* **73**, 121–125 (2017).

[34] Šimko, F. *et al.* Cesium Oxo-fluoro-aluminates in the CsF–Al_2O_3 System: Synthesis and Structural Characterization. *Inorganic Chemistry* **62**, 15651–15663 (2023).

[35] Morrison, G., Jones, V. G., Zamorano, K. P., Greedan, J. E. & Zur Loye, H.-C. Flux Synthesis, UV–vis Absorbance, and Magnetism of Cesium Copper Silicates with an Isolated Super-Super Exchange Spin Dimer in $Cs_6Cu_2Si_9O_{23}$. *Inorganic Chemistry* **62**, 11682–11689 (2023).

[36] Kimura, H. & Yamane, H. Crystal structure of chain silicate $Cs_3LuSi_3O_9$. *Acta Crystallographica Section E: Crystallographic Communications* **77**, 1239–1242 (2021).

[37] Craig, A. J., Stoyko, S. S., Bonnoni, A. & Aitken, J. A. Syntheses and crystal structures of the quaternary thiogermanates $Cu_4FeGe_2S_7$ and $Cu_4CoGe_2S_7$. *Acta Crystallographica Section E: Crystallographic Communications* **76**, 1117–1121 (2020).

[38] Zhang, N. *et al.* $Eu_2MGe_2OS_6$ (M= Mn, Fe, Co): Three Melilite-Type Rare-Earth Oxythiogermanates Exhibiting Balanced Nonlinear-Optical Behaviors. *Inorganic Chemistry* **62**, 16299–16303 (2023).

[39] Huang, Y. *et al.* HgB_2S_4: A d^{10} Metal Thioborate with Giant Birefringence and Wide Band Gap. *Chemistry of Materials* (2023).

[40] Ramakrishnan, S. *et al.* Coupling between Charge Density Wave Ordering and Magnetism in $Ho_2Ir_3Si_5$. *Chemistry of Materials* **35**, 1980–1990 (2023).

[41] Redhammer, G. J. & Tippelt, G. The crystal structure of $KScP_2O_7$. *Acta Crystallographica Section E: Crystallographic Communications* **76**, 1412–1416 (2020).

[42] Dong, W. *et al.* $K_2Sr_4(PO_3)_{10}$: A Polyphosphate with Deep-UV Cutoff Edge and Enlarged Birefringence. *Inorganic Chemistry* **62**, 16215–16221 (2023).

[43] Eder, F. & Weil, M. Crystal structure of $K_6[Zn(CO_3)_4]$. *Acta Crystallographica Section E: Crystallographic Communications* **79**, 718–721 (2023).

[44] Yan, H. *et al.* $La_4Ga_2S_8O_3$: A Rare-Earth Gallium Oxysulfide with Disulfide Ions. *Inorganic Chemistry* (2023).

[45] Zhang, H. *et al.* Prediction and Synthesis of a Selenide Perovskite for Optoelectronics. *Chemistry of Materials* (2023).

[46] Pavlyuk, V., Dmytriv, G., Tarasiuk, I. & Ehrenberg, H. $Li_9Al_4Sn_5$ as a new ordered superstructure of the $Li_{13}Sn_5$ type. *Acta Crystallographica Section C: Structural Chemistry* **73**, 337–342 (2017).

[47] Nishita, Y., Simura, R., Inaguma, Y. & Yamane, H. $LiBa_2AlO_4$: A new lithium barium aluminate having an oxygen tetrahedral framework. *Journal of Solid State Chemistry* **317**, 123654 (2023).

[48] Roh, J., Do, N., Manjón-Sanz, A. & Hong, S.-T. Li_2GeS_3: Lithium Ionic Conductor with an Unprecedented Structural Type. *Inorganic Chemistry* **62**, 15856–15863 (2023).

[49] Gvozdetskyi, V. *et al.* From Layered Antiferromagnet to 3D Ferromagnet: LiMnBi-to-MnBi Magneto-Structural Transformation. *Chemistry of Materials* **35**, 3236–3248 (2023).

[50] Hyde, P. *et al.* Lithium Intercalation into the Excitonic Insulator Candidate Ta_2NiSe_5. *Inorganic Chemistry* **62**, 12027–12037 (2023).

[51] Ponou, S., Lidin, S. & Mudring, A.-V. Optimization of Chemical Bonding through Defect Formation and Ordering–The Case of $Mg_7Pt_4Ge_4$. *Inorganic Chemistry* **62**, 8519–8529 (2023).

[52] Kamutzki, F. *et al.* $NaGdSi_2O_6$ – A novel antiferromagnetically coupled silicate with *Vierer* chain structure. *Journal of Solid State Chemistry* **317**, 123677 (2023).

[53] Nagai, T. & Kimura, T. Chemical Switching of Ferroaxial and Nonferroaxial Structures Based on Second-Order Jahn–Teller Activity in $(Na\,K)_2Hf(BO_3)_2$. *Chemistry of Materials* **35**, 4109–4115 (2023).

[54] Galven, C. *et al.* $Na_6Li_4MO_4(CO_3)_4$ (M= W and Mo): An Alternative Electrolyte for High-Temperature Electrochemical Cells. *Inorganic Chemistry* **62**, 15367–15374 (2023).

[55] Hughes, J. M., Wise, W. S., Gunter, M. E., Morton, J. P. & Rakovan, J. Lasalite, $Na_2Mg_2[V_{10}O_{28}]\cdot 20\,H_2O$, a new decavanadate mineral species from the Vanadium Queen Mine, La Sal District, Utah: Description, atomic arrangement, and relationship to the pascoite group of minerals. *The Canadian Mineralogist* **46**, 1365–1372 (2008).

[56] Luo, Q., Li, N., Zhao, Z., Cui, M. & He, Z. A new compound $Na_5Mn_4(PO_4)_4F_4\cdot 2\,H_2O$ with a rarely mixed valence spin chain showing multiple magnetic transitions. *Inorganic Chemistry Frontiers* **10**, 6303–6307 (2023).

[57] Robert, R., Mangalassery, S., Rao, D. N. & Vidyasagar, K. Syntheses and characterization of quaternary selenites and tellurite of antimony, $NaSbSe_2O_7$, $AgSbSe_2O_7$ and $Na_2Sb_4Te_2O_{14}$. *Journal of Solid State Chemistry* **327**, 124228 (2023).

[58] Novikov, S. *et al.* $Na_{4-x}Sn_{2-x}Sb_xGe_5O_{16}$, an Air-Stable Solid-State Na-Ion Conductor. *Inorganic Chemistry* **62**, 16068–16076 (2023).

[59] Eder, F. & Weil, M. Garnet-type $Na_3Te_2(FeO_4)_3$. *Acta Crystallographica Section E: Crystallographic Communications* **79** (2023).

[60] Chong, S., Kroll, J. O., Crum, J. V. & Riley, B. J. Synthesis and crystal structure of a neodymium borosilicate, $Nd_3BSi_2O_{10}$. *Acta Crystallographica Section E: Crystallographic Communications* **75**, 700–702 (2019).

[61] Eder, F. & Weil, M. $Ni_3Te_2O_2(PO_4)_2(OH)_4$, an open-framework structure isotypic with $Co_3Te_2O_2(PO_4)_2(OH)_4$. *Acta Crystallographica Section E: Crystallographic Communications* **76**, 625–628 (2020).

[62] Badri, A., Bembli, M., Alvarez-Serrano, I., López, M. L. & Amara, M. B. Synthesis, single crystal structure, optical and magnetic properties of a new rubidium nickel iron phosphate $RbNiFe(PO_4)_2$. *Journal of Solid State Chemistry* 124141 (2023).

[63] Huang, D. *et al.* Metal Halide Single Crystals $RbCdCl_3$: Sn^{2+} and Rb_3SnCl_7 with Blue and White Emission Obtained via a Hydrothermal Process. *Inorganic Chemistry* **62**, 15943–15951 (2023).

[64] Cherif, F.-Z. *et al.* Crystal structure of $SrCo_4(OH)(PO_4)_3$, a new hydroxyphosphate. *Acta Crystallographica Section E: Crystallographic Communications* **76**, 1022–1026 (2020).

[65] Hyoung, J., Lee, H. W., Kim, S. J., Shin, H. R. & Hong, S.-T. Crystal structure of strontium perchlorate anhydrate, $Sr(ClO_4)_2$, from laboratory powder X-ray diffraction data. *Acta Crystallographica Section E: Crystallographic Communications* **75**, 447–450 (2019).

[66] Menezes, L. T. *et al.* $Sr_6Ge_3OSe_{11}$: A Rationally Designed Noncentrosymmetric Oxyselenide with Polar [$GeOSe_3$] Building Blocks. *Chemistry of Materials* **35**, 3033–3040 (2023).

[67] Xie, Y. *et al.* A series of new rare-earth sulfide borates $RE_3S_3BO_3$ (RE= Nd, Tb, Dy): Syntheses, structures and optical properties. *Journal of Solid State Chemistry* **327**, 124277 (2023).

[68] Zhou, C. & Li, R. Large Difference in Nonlinear Optical Activity of Rare Earth Ion Substitution of Bi^{3+} in A_3TeBO_9 (A= Bi, La, Pr, Nd, Sm-Dy). *Inorganic Chemistry* **62**, 11265–11270 (2023).

[69] Mazet, T., Welter, R. & Malaman, B. A study of the new ferromagnetic $YbMn_6Sn_6$ compound by magnetization and neutron diffraction measurements. *Journal of Magnetism and Magnetic Materials* **204**, 11–19 (1999).

[70] Eder, F. & Weil, M. Crystal structure of $Zn_2(HTeO_3)(AsO_4)$. *Acta Crystallographica Section E: Crystallographic Communications* **77**, 555–558 (2021).

[71] Hu, C.-L., Han, Y.-X., Fang, Z. & Mao, J.-G. Zn_2BS_3Br: An Infrared Nonlinear Optical Material with Significant Dual-Property Enhancements Designed through a Template Grafting Strategy. *Chemistry of Materials* **35**, 2647–2654 (2023).

[72] Santamaría-Pérez, D., Chuliá-Jordán, R., Otero-de-la Roza, A., Oliva, R. & Popescu, C. High-Pressure Experimental and DFT Structural Studies of Aurichalcite Mineral. *Minerals* **13**, 619 (2023).

[73] Jiao, R. *et al.* Crystal Structure Prediction by Joint Equivariant Diffusion. *arXiv preprint arXiv:2309.04475* (2023).

[74] Jiao, R., Huang, W., Liu, Y., Zhao, D. & Liu, Y. Space Group Constrained Crystal Generation. *arXiv preprint arXiv:2402.03992* (2024).

[75] Yang, M. *et al.* Scalable Diffusion for Materials Generation. *arXiv preprint arXiv:2311.09235* (2023).

[76] Flam-Shepherd, D. & Aspuru-Guzik, A. Language models can generate molecules, materials, and protein binding sites directly in three dimensions as XYZ, CIF, and PDB files. *arXiv preprint arXiv:2305.05708* (2023).

[77] Gruver, N. *et al.* Fine-Tuned Language Models Generate Stable Inorganic Materials as Text. *arXiv preprint arXiv:2402.04379* (2024).

[78] Van der Maaten, L. & Hinton, G. Visualizing Data using t-SNE. *Journal of Machine Learning Research* **9** (2008).
