# Supplementary material for: Crystal structure generation with autoregressive large language modeling
Source: Nat Commun. 2024 Dec 6;15:10570. doi: 10.1038/s41467-024-54639-7 (PMC11624194; doi:10.1038/s41467-024-54639-7)
Supplement: Supplementary file 2 — Description of Additional Supplementary Files [file 41467_2024_54639_MOESM2_ESM.pdf]

## Description of Additional Supplementary Files:

**Supplementary Data 1:** A CSV file containing the $E_{hull}$ values of the 102 compounds identified as novel after 1,000 unconditional generation attempts using the model. The file contains the following columns: "Compound", which contains an index identifying a compound; "E hull orig DFT", the DFT-based above-hull energy of the original compound; "E hull MCTS DFT", the DFT-based above-hull energy of the compound after MCTS; "Delta E DFT", the difference between the DFT-based above-hull energies of the compound after MCTS and before; "E ALIGNN orig", the ALIGNN energy of the original compound; "E ALIGNN MCTS", the ALIGNN energy of the compound after MCTS; "Delta E ALIGNN", the difference between the ALIGNN energies of the compound after MCTS and before. All energies are in eV/atom.
